# Local-scale models reveal ecological niche variability in amphibian and reptile communities from two contrasting biogeographic regions

**DOI:** 10.7717/peerj.2405

**Published:** 2016-10-06

**Authors:** Alberto Muñoz, Xavier Santos, Ángel M. Felicísimo

**Affiliations:** 1 Graphics Engineering, Geomatics and Projects, Department of Graphic Expression, University Center of Merida, University of Extremadura, Mérida, Cáceres, Spain; 2 Research Centre in Biodiversity and Genetic Resources CIBIO/InBIO, Institute of Agricultural Sciences of Vairão, University of Porto, Vairão, Portugal

**Keywords:** Amphibians, Community, Distribution patterns, Modelling, Maximum entropy modelling, Reptiles

## Abstract

Ecological Niche Models (ENMs) are widely used to describe how environmental factors influence species distribution. Modelling at a local scale, compared to a large scale within a high environmental gradient, can improve our understanding of ecological species niches. The main goal of this study is to assess and compare the contribution of environmental variables to amphibian and reptile ENMs in two Spanish national parks located in contrasting biogeographic regions, i.e., the Mediterranean and the Atlantic area. The ENMs were built with maximum entropy modelling using 11 environmental variables in each territory. The contributions of these variables to the models were analysed and classified using various statistical procedures (Mann–Whitney *U* tests, Principal Components Analysis and General Linear Models). Distance to the hydrological network was consistently the most relevant variable for both parks and taxonomic classes. Topographic variables (i.e., slope and altitude) were the second most predictive variables, followed by climatic variables. Differences in variable contribution were observed between parks and taxonomic classes. Variables related to water availability had the larger contribution to the models in the Mediterranean park, while topography variables were decisive in the Atlantic park. Specific response curves to environmental variables were in accordance with the biogeographic affinity of species (Mediterranean and non-Mediterranean species) and taxonomy (amphibians and reptiles). Interestingly, these results were observed for species located in both parks, particularly those situated at their range limits. Our findings show that ecological niche models built at local scale reveal differences in habitat preferences within a wide environmental gradient. Therefore, modelling at local scales rather than assuming large-scale models could be preferable for the establishment of conservation strategies for herptile species in natural parks.

## Introduction

Ecological niche models (ENMs) describe the realised niches of species, i.e., the subset of fundamental niches where species are restricted due to their interspecific interactions (Austin, 2002). ENMs can also be considered mechanistic analyses of how environmental factors affect the fitness of species in their habitat (Kearney, 2006). Thus, ENM application is a useful procedure to identify suitable habitats for species populations by understanding their ecological requirements (Martínez-Freiría et al., 2008; Soberón & Nakamura, 2009). In this context, a relevant debate is the adequacy of extrapolating ENMs between different territories (Thuiller et al., 2004; Elith & Leathwick, 2009; Saupe et al., 2012; Carneiro et al., 2016). Environmental factors vary especially at large geographic scales, and therefore, species tend to change their topographic positions to compensate the variations in resource availability and climatic conditions (Guisan & Zimmermann, 2000). Thus, habitat suitability can differ across geographic ranges of species, and modelling at large-scales would not be able to provide a real insight into the effects of indirect parameters (e.g., topographic variables) in reduced territories (Osborne & Suárez-Seoane, 2002). Furthermore, the quality of resolution and consistency of predictor variables over large geographic scales depends on availability and source of the environmental data or on computing power (Guisan et al., 2007; Vale, Tarroso & Brito, 2014).

One way to mitigate these large-scale difficulties could be modelling at lower scale (Osborne & Suárez-Seoane, 2002). Low-scale spatial distribution can respond to others factors that describe microhabitat conditions (Vale, Tarroso & Brito, 2014), and therefore, a high resolution of predictor variables is important in these models (Mac Nally et al., 2004). However, limitations exist also for low-scale procedures. For example, biotic interactions, which are generally obviated in modelling procedures, have an important effect only at fine scale (Pearson & Dawson, 2003). Thus, the study-area extent can be a critical factor for modelling species distribution, and it can depend on which species are modelled (Wiens, 2002). For example, ENMs in large spatial scales of species inhabiting a wide range of environmental conditions are considered less reliable than those of restricted distribution species because, for a limit sampling data, the range of values of each variable range is higher (Mateo, Felicísimo & Muñoz, 2011). In contrast, working with edge-species, i.e., species associated with the perimeter of a habitat patch (Imbeau, Drapeau & Mönkkönen, 2003), can provide a clearer view of the environmental factors that determine their distribution, because modelling without part of the range border is insufficient (Arntzen, 2006). The ecological niche of species at range limits may differ from that at the species’ complete range (Braunisch et al., 2008), especially if transition zones are involved, because species tend to select narrow conditions (Vale, Tarroso & Brito, 2014). Therefore, local models within range limits of species may be preferred over modelling at a higher scale.

In contrast to other terrestrial vertebrates, amphibians and reptiles are small ectothermic animals that are very sensitive to environmental and habitat attributes and have low dispersal capabilities (García-París, Montori & Herrero, 2004; Salvador, 2014). These features make them useful groups for studying how environmental factors shape their distribution at a local scale (Soares & Brito, 2007; Martínez-Freiría et al., 2008; Couturier et al., 2014). Thus, amphibians and reptiles can be adequate model groups to examine how opposing environmental features can shape the construction of ENMs. Amphibians and reptiles have different ecological requirements, e.g., water for amphibian breeding and solar radiation for reptile physiology. For this reason, both taxonomic classes may be differently influenced by environmental factors (Rodríguez, Belmontes & Hawkins, 2005). As amphibians and reptiles are highly threatened (Baillie, Hilton-Taylor & Stuart, 2004; Cox, Chanson & Stuart, 2006; Carvalho et al., 2010), ENMs have been applied to conservation issues of both taxonomic groups (Gustafson, Murphy & Crow, 2001; Santos et al., 2006; Puschendorf et al., 2009; Couturier et al., 2014). Because ENMs provide valuable information to address conservation issues (Mateo, Felicísimo & Muñoz, 2011), application of local-scale ENMs to amphibian and reptile species can be useful to delineate conservation programs of endangered species/populations.

We have built ENMs of amphibian and reptile communities in two Spanish National Parks, Picos de Europa and Cabañeros, located in two contrasted biogeographic regions of the Iberian Peninsula, the Atlantic and Mediterranean provinces (Rivas-Martínez et al., 2002). The Mediterranean region is characterised by sclerophyllous vegetation adapted to a dry and hot summer, while the Atlantic region is dominated by deciduous forest with mild temperatures and abundant rainfall throughout the year. Comparing ENMs from both areas gives us the opportunity to test to what extent amphibian and reptile species could show different responses in preserved areas located on two contrasted biogeographic regions. Due to the environmental differences between the two studied national parks, we expect that ecogeographical variables will contribute differently to ENMs in both study areas and for both taxonomic groups. For example, the scarcity and unpredictability of water in the Mediterranean region suggest a higher contribution of this variable in Cabañeros, and for amphibian species (due to their dependence of water for their reproduction; Carey & Alexander, 2003). Both study areas differ in altitudinal range and therefore in environmental heterogeneity (higher in Picos de Europa). Thus, we expect that climatic and topographic variables showing a gradient related to altitude could have a higher contribution on performing ENM in Picos de Europa. In regard to the location of both national parks, the modelled species were classified as Mediterranean or Atlantic according to the proportion of their ranges within each biogeographic region (Sillero et al., 2009). Recent studies have shown that this classification results in opposing responses to environmental factors and disturbances (Ferreira, Mateus & Santos, 2016; Ferreira, Žagar & Santos, 2016); indeed, we expect that variable contribution to ENMs will be influenced by the biogeographic affinity of the studied species. As some species are present in both territories, we expect different inter-park responses to environmental factors, particularly those species located outside their dominant biogeographical area or at a distribution limit. These species are an opportunity to strengthen the idea that species do not maintain their realised niche across their entire range of distribution (Braunisch et al., 2008); consequently, ENMs should not be extrapolated between both parks.

In summary, our general objective is to build local-scale ENMs of amphibian and reptile species in two contrasted environmental sites and to understand what variables are more relevant for describing their ecological niches. Specifically, we have addressed the following issues: (1) how does dependency between species or communities and environmental variables vary between the two national parks; (2) how does dependency between species or communities and environmental variables vary between taxonomic classes; (3) what is the variability of species-specific responses to environmental variables; (4) are there differences in ecological niche models for the species present in both territories?

## Materials and Methods

### Study area

**Figure 1:**
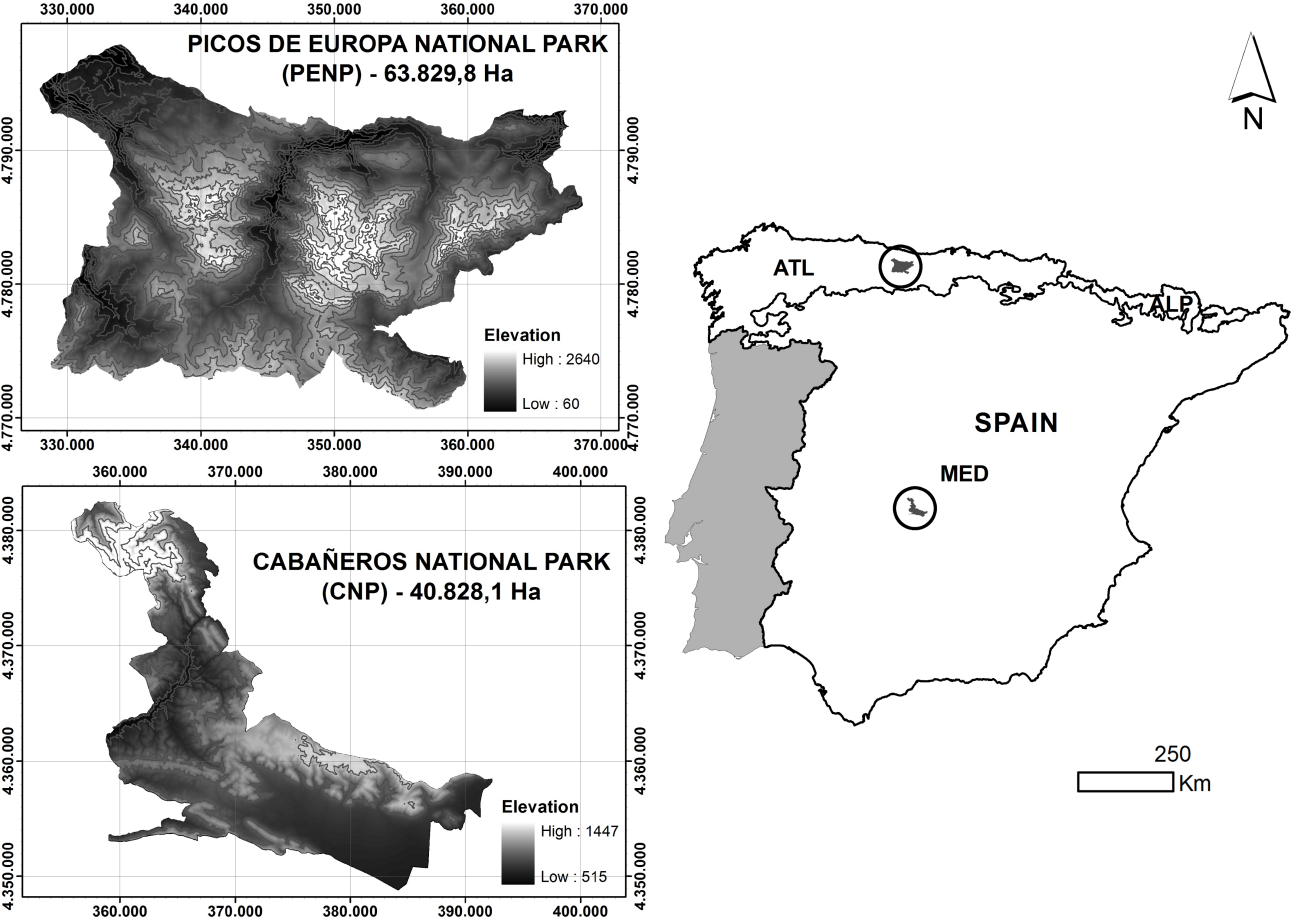
Location and altitudinal range of studied national parks. Biogeographic regions are represented in the Spanish peninsular territory: MED, Mediterranean; ATL, Atlantic; ALP, Alpine.

The two study sites are Cabañeros National Park (hereafter referred to as CNP) and Picos de Europa National Park (hereafter referred to as PENP), which are situated in the Iberian-Western-Mediterranean and Atlantic-European provinces, respectively (Rivas-Martínez et al., 2002), (Fig. 1). CNP is located in the middle of the Iberian Peninsula and south of the Toledo Mountains and covers an area of approximately 408 km^2^. This protected area is completely under a Mediterranean climate of continental influence, defined by a dry summer season with less than 50 mm of average precipitation in this season and high temperatures (34°C mean maximum temperature in July). Its topography is adjusted to the apalachense relief, which lacks significant slopes and altitudes. Most of the territory is located below 1,000 m and consists of vast plains filled with eroded material called “rañas.” The tallest point in the Northwest of the park has an altitude of 1,448 m and consists of Paleozoic mountains in east–west alignment. CNP is mainly composed of Mediterranean forest characterised by a community of sclerophyllous species, mainly holm oak (*Quercus ilex* L.) and cork oak (*Quercus suber* L.). A complex mosaic of vegetation (open tree land, scrubland and grassland) coexists with the Mediterranean forest due to long-term human activity in the area (Vaquero de la Cruz, 1997).

PENP is situated north of the Iberian Peninsula and occupies an extension of approximately 638 km^2^. The dominant climate is Atlantic, with marginal Alpine and Mediterranean influences on mountain tops and in lowland areas, respectively. Most of the territory has an annual precipitation of more than 1,300 mm/year, particularly in the northern mountains. Its topography is formed by three mountainous massifs (Western, Central and Eastern) in the middle of the Cantabrian Mountains. In contrast to Cabañeros, there is a marked variation in environmental conditions due to a wide altitudinal gradient from 48 m to 2,460 m. Consequently, PENP has a wide representation of habitats. Grassland occurs on lowland pastures (*Cynosurus cristatus*), medium altitude slopes and subalpine meadows on the highest parts of the mountains (*Nardus stricta* and *Festuca burnatii*). There is a large representation of scrublands (*Genista spp., Cytisus spp., Erica spp., Calluna spp*.). Deciduous forests on valley bottoms and mid-slopes are dominated by beech (*Fagus sylvatica*). Riparian forest (*Alnus glutinosa*) is confined to valley bottoms, and prickly juniper (*Juniperus oxycedrus*) and holm oak woodlands (*Quercus rotundifolia*) are restricted to the exposed slopes of limestone gorges. Finally, there are fragmented stands of conifer plantations.

### Data collection

**Figure 2:**
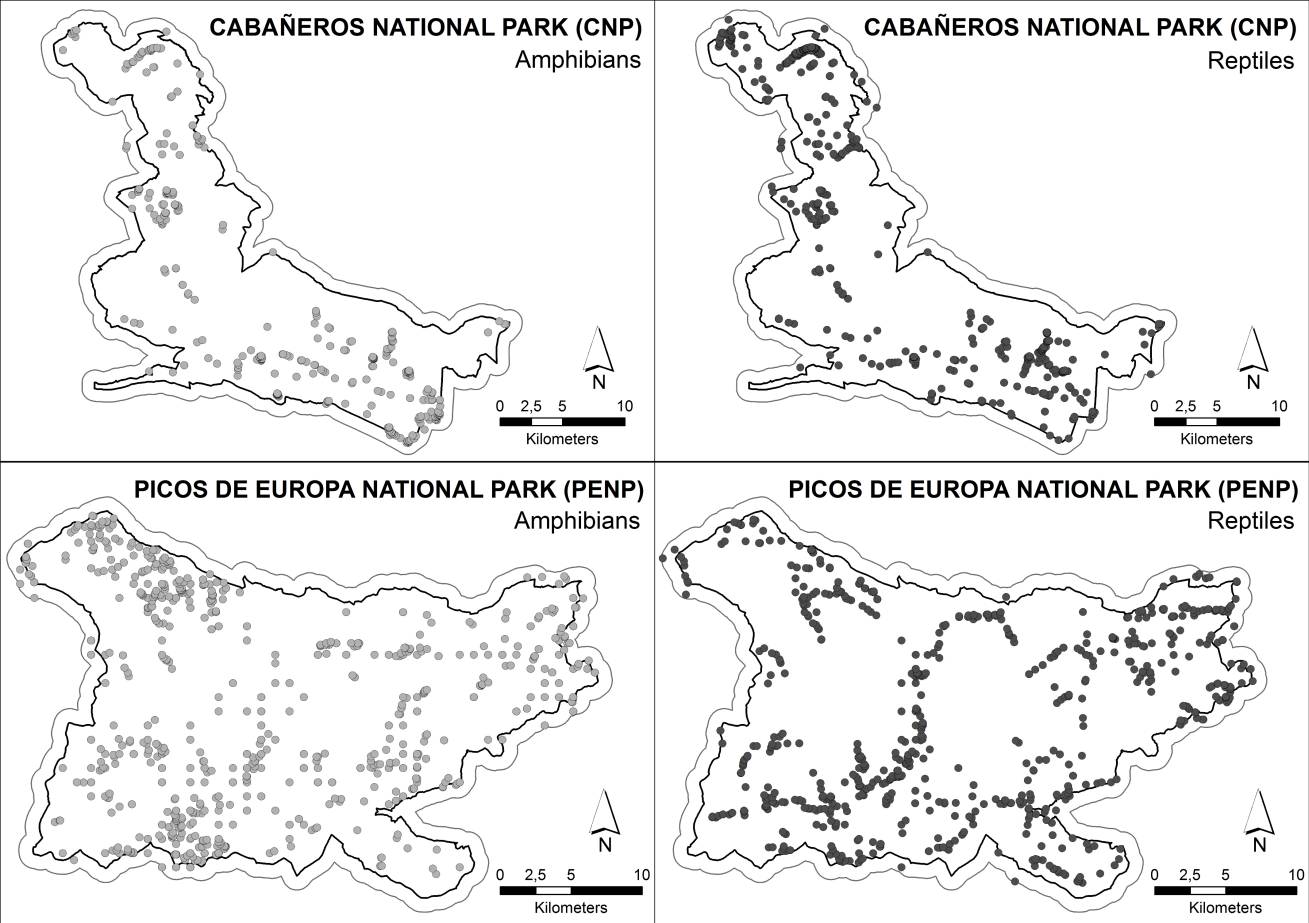
Distribution of the species’ citations used into MaxEnt models. Boundary less saturated line indicates the buffer of 1 km which was made to include records located in the immediate boundaries.

The data set for building ENMs was composed of 529 and 662 exact locations of 12 amphibian and 18 reptile species, respectively, in CNP and 1,108 and 1,196 exact locations of 9 amphibian and 12 reptile species, respectively, in PENP (Fig. 2). We considered a location as an observation of one specific species with accuracy of less than 1 × 1 m, regardless of the number of individuals observed. These locations were obtained from standardised fieldwork protocols covering both study areas between 2005 and 2008 in PENP and between 2008 and 2010 in CNP. These territories were divided into grids of cells of 2 × 2 km as mapping units to ensure proper representation. Fieldwork was conducted by herpetological experts through active searches in suitable habitats and under optimal weather conditions for each taxonomic family. The fieldwork was coordinated by the Asociación Herpetológica Española (AHE) under different projects which aimed at creating herpetological atlases of both national parks. Additionally, we used records opportunistically collected between 1990 and 2012 from other sources (e.g., databases of both national parks and records stored in the AHE Spanish database). Finally, a buffer zone of 1 km was created for each national park to include records located in the immediate park boundaries in order to increase the data set of species with marginal distribution in the national parks, which resulted in a substantial improvement of the models.

### Environmental variables

We used a set of 48 ecogeographical variables (EGVs) to build ENMs grouped into the following categories: climate, topography, potential solar radiation, vegetation and hydrology (Table 1). These EGVs are relevant to model amphibian and reptile niches (Arntzen, 2006; Sillero et al., 2009; Urbina-Cardona & Flores-Villela, 2010). For example, as amphibians and reptiles are ectothermic vertebrates, their distribution is strongly constrained by temperature and precipitation (Aragón et al., 2010). Amphibians highly depend on water availability; thus, a high impact of the hydrological network is expected (Jakob et al., 2003). Solar exposure is a common feature in explaining many aspects of reptile life-history, therefore, it might be important to consider energy input and terrestrial habitat in models (Sillero et al., 2009; Pike, Webb & Shine, 2011). Altitude and slope are common factors used in analysing spatial distribution of herptiles (Sá-Sousa, 2000; Guisan & Hofer, 2003; Soares & Brito, 2007).

**Table 1:** Environmental variables. Abbreviations are shown only for environmental variables were used into the last MaxEnt models, in other case the variables appear as “no retained”.

Variables	Units	Abbreviation
CLIMATOLOGY		
Annual mean precipitation	mm	AP
Minimum mean temperature of the coldest month (January)	°C	MnT
Maximum mean temperature of the hottest month (July)	°C	MxT
Mean precipitation of each month	mm	No retained
Minimum mean temperature of each month	°C	No retained
Maximum mean temperature of each month	°C	No retained
TOPOGRAPHY		
Altitude a.s.l.	m	DEM_A
Slope	°	DEM_S
INSOLATION		
Solar radiation in spring equinox	kJ/m^2^	SRSE
Solar radiation in summer solstice	kJ/m^2^	SRSS
Solar radiation in winter solstice	kJ/m^2^	No retained
Solar radiation in intermediate days	kJ/m^2^	No retained
VEGETATION		
Vegetation structure (land uses)	*14 categories*	VS
Spatial distribution of tree vegetation	*7 categories*	TreeV
Fraction of canopy cover	%	FCC
HYDROLOGY		
Distance to the hydrological network	m	DHN
Distance to temporary water bodies	m	No retained

To create climatic variables, we used a climatic series constructed by the research group Kraken (University of Extremadura) and applying the method of kriging on specific data of weather stations, transferred by the State Agency of Meteorology (Government of Spain) between 1971–2007. Altitude, slope and radiation variables were obtained from a Digital Terrain Model with a mesh size of 5 m; the data is available on the website of the National Centre for Geographic Information (Government of Spain). Radiation variables were calculated using algorithms which estimate both direct and diffuse radiation (Kumar, Skidmore & Knowles, 1997). Vegetation variables were obtained by rasterization of forestry Maps (1:50,000) of the Ministry of Agriculture, Food and Environment (Government of Spain), using information about structure types, distribution of vegetation and woody canopy cover. Hydrographic elements may constitute potential habitats for amphibian and some reptile species and were extracted from the layers of the National Topographic Database (National Centre for Geographic Information, Government of Spain). Finally, these layers were treated in a large geographic processing order to create distance to the hydrological network. Environmental layers and georeferenced data were managed using Geographic Information Systems (ArcGIS and ArcInfo, ESRI Inc.). The grain size of layers was 5 × 5 m to provide a high resolution of the predictive variables.

### Modelling procedures

The maximum entropy modelling or MaxEnt model (Phillips, Anderson & Schapire, 2006) was selected to determine the relative importance of each environmental variable for modelling habitat suitability areas. First, we constructed models using all potential variables to create a complete picture of how each variable influences the models. Thus, before making a selection, we identified the most explanatory EGVs by analysing their response curves and percentages of contribution. As a general rule, we discarded variables with a high linear correlation between them (*r* > 0.75), using loosely correlated environmental layers (Table S1), and finally retained 11 variables to run the models. Variables related to temperature and radiation were highly correlated, and we selected those with greater explanatory in previous MaxEnt models. Thus, we retained the temperature variables with more extreme values (the coldest month and the hottest month) and the radiation variables with higher contrast values (i.e., higher standard deviation and range between maximum and minimum). Within the variables related to precipitation, we retained annual rainfall.

The retained variables (Table 1) were used to run the models separately for each study area in two steps. In the first step, the option “random test percentage” was checked (with a value of 20%) to withhold a percentage of the presence data to evaluate the model’s performance. In the second step, the models were repeated including all the presence data. The contribution of EGVs to the models was calculated in percentages as follows: in each iteration of the training algorithm, the increase in regularised gain is added to the contribution of the corresponding variable or subtracted from it if the change to the absolute value of lambda is negative (Phillips, Anderson & Schapire, 2006). This statistical procedure allows us to have a non-metric value to compare different environmental factors. Other outputs, i.e., response curves and jackknife tests of variable importance, were checked as useful references to interpret the models.

### Data analysis

The contribution of environmental variables to the models was used to examine the variation in the ecological niche between both taxonomic groups and biogeographic affinities of species within the two study areas. The statistical analysis was conducted in three steps: (1) Mann–Whitney *U* tests (due to non-normal distribution of inputs) were used to check for differences of the contribution of each EGV between taxonomic groups and biogeographic regions at both study areas; (2) Principal Components Analysis (PCA) with all EGVs contribution was performed to reduce the variance of these variables into a set of main components. Additionally, the VARIMAX rotation was used to minimise the number of variables with high loadings on each factor; (3) the values of PCA axes 1 and 2 for each species were examined by General Linear Models using taxonomic group, national park and biogeographic affinity and their interactions as factors. Biogeographic affinity was established, according to the classification of each amphibian/reptile species into their main geoclimatic regions (Sillero et al., 2009), as Mediterranean (the percentage of inclusion of the habitat suitability in Mediterranean region is more than 50%) or non-Mediterranean (the percentage of inclusion of the habitat suitability in Mediterranean region is less than 50%).

## Results

### Contribution of environmental variables to the models

Distance to the hydrological network was consistently the most relevant variable for both parks and taxonomic classes (Table 2), although distance to temporary water bodies was discarded due its low contribution to models (Table 1). The contribution of distance to the hydrological network exceeded 10% in 76.5% of the models and was higher in CNP than in PENP in both amphibian and reptile species (Tables 2 and 3). The topographic variables slope and altitude occasionally had very high contributions (maximum close to 70%), and their roles were transposed by the taxa: the contribution of slope was higher for amphibians and altitude dominated for reptiles (Tables 2 and 3). Contribution of minimum mean temperature of the coldest month reached the highest values in PENP, while contribution of maximum temperature of the hottest month and annual mean precipitation reached the highest values in CNP (Tables 2 and 3). Regarding vegetation variables, vegetation structure had the higher values of contributions to models (Table 2), while the fraction of canopy cover and, specifically, spatial distribution of tree vegetation exceed 10% in contributing to the models only in isolated cases. Finally, solar radiation had a significant contribution for a few species. Reptiles in PENP were subjected to the highest values of solar radiation at the time of spring equinox (Tables 2 and 3). It should also be noted that, in addition to these results, a high variability of variable contribution remains at the species level within both parks and taxonomic classes (Tables S2 and S3).

**Table 2:** Contributions of environmental variables to the MaxEnt models. *N* = Number of total species models. Median (above), minimum and maximum (below) are shown for each variable. Variable abbreviations are detailed in Table 1.

		DHN	DEM_S	DEM_A	MnT	VS	AP	MxT	SRSS	FCC	SRSE	TreeV
**CNP**	Amphibians	38.8	20.1	4.9	1.0	5.6	5.4	3.3	1.7	1.6	0.3	0.0
	*N* = 12	3.1–78.0	0.0–57.0	0.0–27.3	0.0–24.0	0.5–41.7	0.0–14.5	0.0–17.6	0.0–21.9	0.0–15.7	0.0–2.1	0.0–3.6
	Reptiles	34.4	3.6	2.6	6.0	5.0	7.3	2.0	1.4	0.2	0.3	0.0
	*N* = 18	0.0–80.3	0.0–68.7	0.0–25.6	0.0–55.7	0.4–44.4	0.0–53.3	0.0–15.1	0.0–28.9	0.0–20.5	0.0–2.6	0.0–20.0
	Total	**37.2**	**5.7**	**3.3**	**3.9**	**5.0**	**6.4**	**2.8**	**1.7**	**0.7**	**0.3**	**0.0**
		0.0–80.3	0.0–68.7	0.0–27.3	0.0–55.7	0.4–44.4	0.0–53.3	0.0–17.6	0.0–28.9	0.0–20.5	0.0–2.6	0.0–20.0
**PENP**	Amphibians	20.2	28.4	7.4	9.0	2.7	7.4	2.1	1.5	1.2	1.5	0.6
	*N* = 9	0.0–37.8	0.1–52.6	0.4–69.3	4.3–59.8	0.2–21.8	0.0–16.0	0.0–5.3	0.0–4.7	0.0–16.2	0.0–3.8	0.0–8.5
	Reptiles	11.0	10.3	25.8	7.3	4.1	0.7	2.0	1.3	1.1	7.2	0.3
	*N* = 12	3.5–63.9	0.0–48.7	3.2–61.1	0.0–28.3	0.0–15.8	0.0–5.1	0.0–10.4	0.0–16.7	0.0–7.4	0.0–11.9	0.0–14.2
	Total	**15.3**	**11.8**	**15.1**	**8.1**	**2.9**	**0.9**	**2.1**	**1.4**	**1.2**	**2.8**	**0.3**
		0.0–63.9	0.0–52.6	4.0–69.3	0.0–59.8	0.0–21.8	0.0–16.0	0.0–10.4	0.0–16.7	0.0–16.2	0.0–11.9	0.0–14.2
**TOTAL**	Amphibians	**21.3**	**23.8**	**6.7**	**4.4**	**4.4**	**6.8**	**3.1**	**1.7**	**1.2**	**0.7**	**0.0**
	*N* = 21	0.0–78.0	0.0–57.0	0.0–69.3	0.0–59.8	0.2–41.7	0.0–16.0	0.0–17.6	0.0–21.9	0.0–16.2	0.0–3.8	0.0–8.5
	Reptiles	**23.7**	**5.4**	**11.9**	** 6.4**	**4.6**	**2.7**	**2.0**	**1.4**	** 0.3**	**0.7**	**0.1**
	*N* = 30	0.0–80.3	0.0–68.7	0.0–61.1	0.0–55.7	0.0–44.4	0.0–53.3	0.0–15.1	0.0–28.9	0.0–20.5	0.0–11.9	0.0–20.0
	**Total**	**22.5**	**7.9**	** 7.4**	**5.5**	**4.4**	**4.0**	**2.2**	** 1.5**	**0.9**	**0.7**	**0.0**
		0.0–80.3	0.0–68.7	0.0–69.3	0.0–59.8	0.0–44.4	0.0–53.3	0.0–17.6	0.0–28.9	0.0–20.5	0.0–11.9	0.0–20.0

**Table 3:** *U* of Mann–Whitney tests. Comparisons made for: (1) Cabañeros National Park and Picos de Europa National Park (CNP and PENP); (2) both taxonomic classes (Amphibia and Reptilia); (3) Amphibians and Reptiles of Cabañeros National Park (Amphibia/CNP and Reptilia/CNP); (4) Amphibians and Reptiles of Picos de Europa National Park (Amphibia/PENP and Reptilia/PENP); (5) Amphibians of Cabañeros National Park and Amphibians of Picos de Europa National Park (Amphibia/CNP and Amphibia/PENP); (6) Reptiles of Cabañeros National Park and Reptiles of Picos de Europa National Park (Reptilia/CNP and Reptilia/PENP). *P* values <0.05 are in bold type and *P* values >0.1 are indicated as N.S. (non-significant). In brackets, for *P* values <0.1, it is indicated the group with the highest average in the contribution of each variable. Variable abbreviations are detailed in Table 1.

	DHN	DEM_A	DEM_S	AP	MnT	MxT	VS	TreeV	FCC	SRSE	SRSS
(A) CNP	**0.026 (A)**	**0.001 (B)**	N.S.	0.059 (A)	**0.027 (B)**	N.S.	**0.026 (A)**	** 0.011 (B)**	N.S.	**0.001 (B)**	N.S.
(B) PENP											
(A) Amphibia	N.S.	N.S.	**0.033 (A)**	N.S.	N.S.	N.S.	N.S.	N.S.	**0.045 (A)**	N.S.	N.S.
(B) Reptilia											
(A) Amphibia/CNP	N.S.	N.S.	N.S.	N.S.	0.089 (B)	N.S.	N.S.	N.S.	0.084 (A)	N.S.	N.S.
(B) Reptilia/CNP											
(A) Amphibia/PENP	N.S.	**0.043 (B)**	N.S.	**0.046 (A)**	N.S.	N.S.	N.S.	N.S.	N.S.	0.059 (B)	N.S.
(B) Reptilia/PENP											
(A) Amphibia/CNP	0.095 (A)	N.S.	N.S.	N.S.	**0.004 (B)**	N.S.	0.055 (A)	0.070 (B)	N.S.	N.S.	N.S.
(B) Amphibia/PENP											
(A) Reptilia/CNP	0.090 (A)	**0.001 (B)**	N.S.	**0.011 (A)**	N.S.	N.S.	N.S.	0.078 (B)	N.S.	**0.002 (B)**	N.S.
(B) Reptilia/PENP											

### Contribution of EGVs between national parks: PCA results

**Figure 3:**
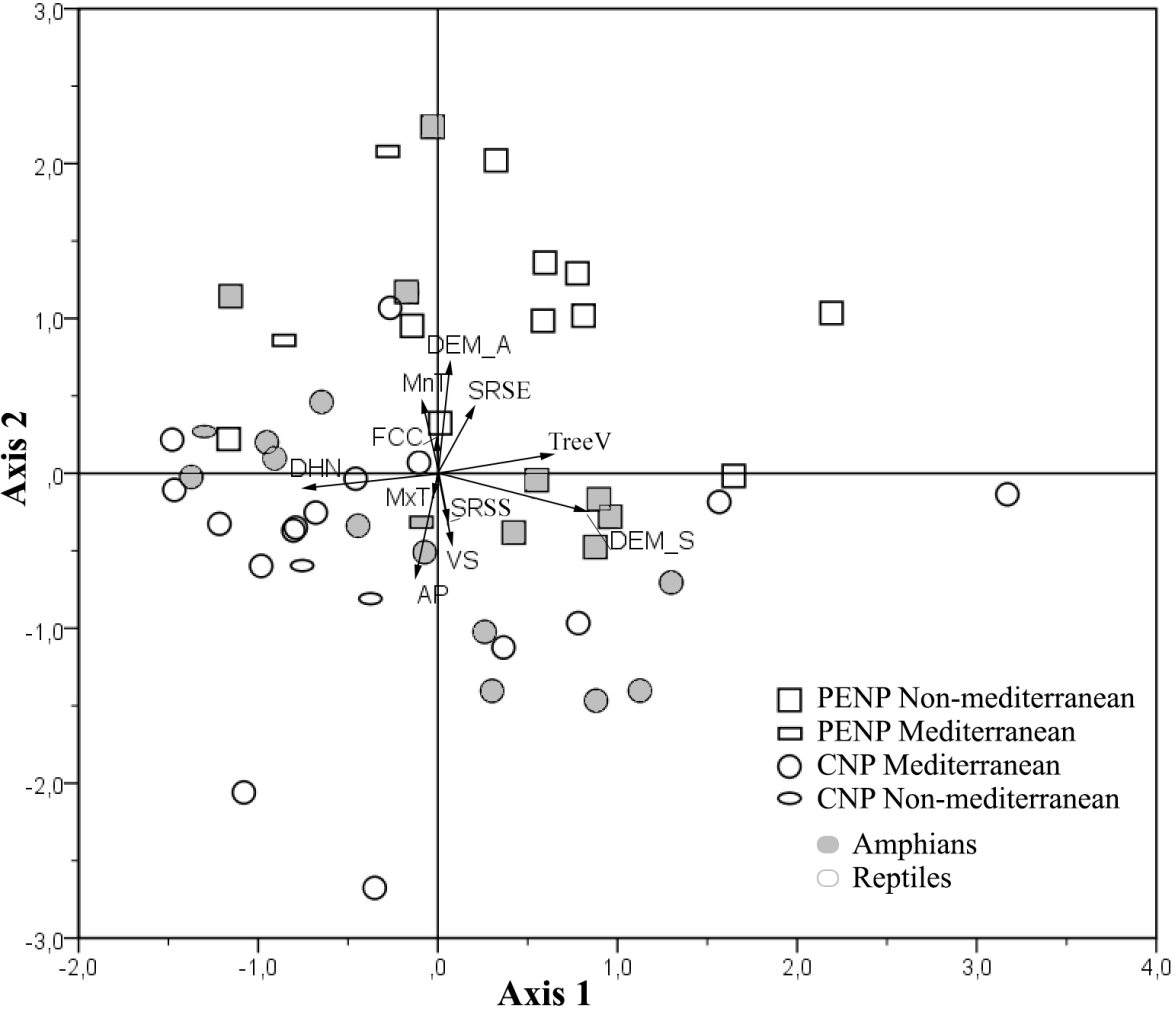
Principal Components Analysis (PCA) of the variable contributions for all species models and variables summarized into two main components. First axis is defined by slope, distance to the hydrological network and spatial distribution of tree vegetation, whereas the second axis is defined by altitude, annual mean precipitation, Minimum mean temperature of the coldest month, vegetation structure and solar radiation in spring solstice. Species models are classified by studied areas (PENP and CNP), Mediterranean affinity and taxonomic groups. Variable abbreviations are detailed in Table 1.

PCA showed a clear discrimination between ENMs from CNP and PENP according to the first and second axis (17.2 and 16.2% explained the variance, respectively; Fig. 3). Models from CNP were grouped in the direction of distance to the hydrological network and annual mean precipitation, and models from PENP were grouped in the direction of altitude and slope in addition to minimum mean temperature of the coldest month. Slope also determined the distribution of some models in CNP (Fig. 3). Therefore, variables related to water availability explain the most variability in CNP species models, and the topography variables with minimum mean temperature of the coldest month were the most relevant in PENP species models. This pattern is enhanced in the case of the species located outside their dominant biogeographical area, i.e., Mediterranean species in PENP and non-Mediterranean species in CNP (Fig. 3). Thus, non-Mediterranean species models (*Salamandra salamandra, Natrix natrix* and *Lacerta schreiberi*) in CNP are associated to the factors distance to the hydrological network, annual mean precipitation or both. Furthermore, the Mediterranean species models (*Natrix maura* and *Timon lepidus*) in PENP are strongly associated with the second component, in the direction of altitude and minimum mean temperature of the coldest month.

**Figure 4:**
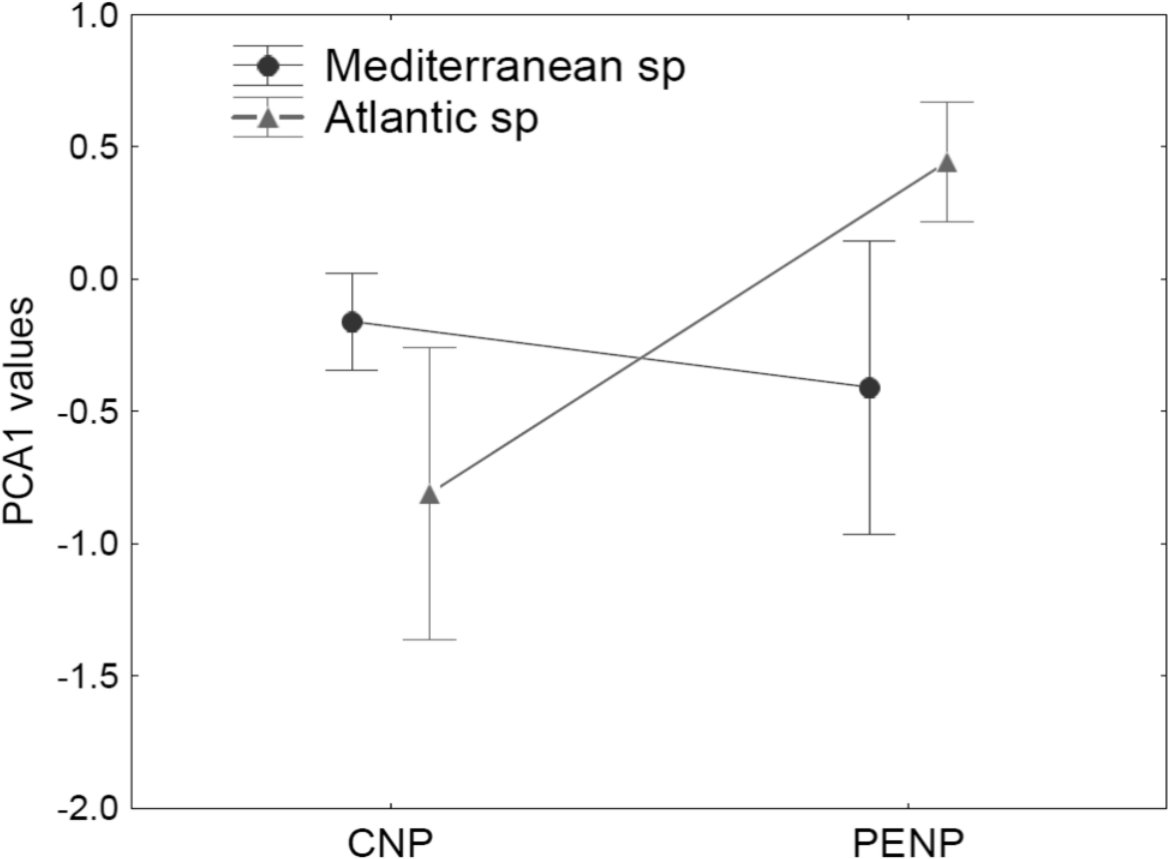
Median and standard error for values of the first PCA axis, analysing interaction between the factors site (CNP and PENP) and biogeographic affinity (Mediterranean and non-Mediterranean). The values of GLMs for this interaction became marginally significant (*F*_1,47_ = 3.23; *P* = 0.08).

Using taxonomy (amphibians and reptiles) and site (CNP and PENP) as factors, GLMs showed only significant differences for site, both for values of the first (marginal differences; *F*_1,47_ = 3.39; *P* = 0.07) and second PCA axis (*F*_1,47_ = 26.43; *P* < 0.001) axis. When we repeated these analyses with site and biogeographic affinity (Mediterranean and non-Mediterranean), the interaction between both became marginally significant for the first axis (*F*_1,47_ = 3.23; *P* = 0.08). Mediterranean species did not show different values for the first PCA axis between both study areas (Fig. 4). In contrast, non-Mediterranean species from CNP had lower values for the first PCA axis than those non-Mediterranean species from PENP, suggesting that these species are more sensitive to the distance to the hydrological network outside their biogeographic domain (i.e., in CNP) (Fig. 4).

### Response of species-specific models to environmental variables

For many studied species, there was a rapid decrease in the probability of occurrence as distance to the hydrological network increased, particularly in CNP (e.g., *Salamandra salamandra*, Fig. S1). Species with greater tolerance to high altitudes were mainly found in PENP, although several species are restricted to lowland areas. With respect to slope, some species, particularly amphibians, have a decreased probability of occurrence as slope increases. In contrast, the probability of occurrence of some species, particularly reptiles, increases as slope increases (e.g., *Tarentola mauritanica*). Likewise, in PENP, amphibians and reptiles have opposing responses to minimum mean temperature of the coldest month, i.e., maximum probability of occurrence at low values for several reptiles and vice versa. The response plots of this variable were in accordance with those of thermopile species such as *Malpolon monspessulanus* and *Hemorrhois hippocrepis*. For vegetation structure, there were species-specific responses according to particular habitat types, for example agrosylvopastoral system on *Chalcides striatus* and wetland on *Zootoca vivipara* (Fig. S2).

Species that inhabit both study areas had opposing response curves for some variables: for example, *Timon lepidus* and *Salamandra salamandra* had a maximum probability of occurrence at different altitudes. The response curves of annual mean precipitation for *Lacerta schreiberi* and *Natrix natrix* and fraction of canopy cover for *Lacerta schreiberi* had a positive slope in CNP, but lacked slope in PENP. Additionally, these species have opposing responses to solar radiation at the time of summer solstice between both study areas. On the other hand, the response curves of *Lacerta schreiberi* and *Salamandra salamandra* (non-Mediterranean species) for their most predictive variables in CNP (i.e., distance to the hydrological network, solar radiation at summer solstice and annual mean precipitation) were more accentuated.

## Discussion

### Contribution of environmental variables to ecological niche models of amphibians and reptiles

In this study, the importance of climate variables for modelling is replaced by other factors, such as altitude, slope and, in particular, the presence of watercourses and ponds. While several studies have reported that climatic variables are the best predictors to explain the ecological niches of amphibians and reptiles at regional or higher geographic scales (Guisan & Hofer, 2003; Rodríguez, Belmontes & Hawkins, 2005; Araújo, Thuiller & Pearson, 2006; Sillero et al., 2009; Urbina-Cardona & Flores-Villela, 2010), our results indicate that at local scale, the importance of climate can be overcome by other non-climate factors (Mateo, Felicísimo & Muñoz, 2011; Real et al., 2013). For example, topographic variables, which can reveal the selection of microhabitats (Guisan & Zimmermann, 2000), play an important role in this study. Our results can also be explained as at low scale, climatic variables can show less prediction capability because the ranges of values for precipitation and temperature are lower.

We found that all the EGV categories were relevant in predicting the potential distribution of species in the following descending order of importance: (1) Hydrology, (2) Topography, (3) Climate, (4) Vegetation and (5) Insolation. The distance to the hydrological network is clearly the variable that most contributes to the distribution models. On the one hand, amphibian breeding is closely linked to freshwater environments. Beyond reproduction, numerous reptiles and most amphibians spend much of their life spans near water (García-París, Montori & Herrero, 2004; Salvador, 2014). In terms of climatic variables, considering that we are modelling ectothermic species, low temperatures should be more decisive in explaining their absence (Moreno-Rueda & Pizarro, 2009; Doody & Moore, 2010), which is reflected in the higher contribution of the minimum mean temperature of the coldest month. One of the vegetation variables (vegetation structure) reflects land use and, consequently, has a major impact on predicting potential distribution.

### Differences between CNP and PENP

The PCA results demonstrated a clear segregation of the contribution of environmental variables to species models between CNP and PENP. Mediterranean climate is characterised by a dry summer with high temperatures. Consequently, there is a pronounced water deficit for at least three months. Despite a moderate influence of continental climate, CNP is completely dominated by Mediterranean features; for this reason, the variables related to water availability (distance to the hydrological network and annual mean precipitation) had, as we expected, the larger contribution to the models in this park. Moreover, human management in CNP has created a landscape mosaic (Vaquero de la Cruz, 1997), and this fact may explain why the contribution of several ENMs in this park were grouped into vegetation structure classes in the PCA (Fig. 3). In contrast to CNP, the predominant climate of PENP is Atlantic, which implies high levels of widespread rain throughout the territory. Additionally, PENP shows a wide altitudinal range and slope (2,412 metres separate the highest and lowest points, and approximately 90% of the land has a slope between 15 and 89°) and, consequently, a wide range of ecological conditions. Therefore, environmental heterogeneity, set in this case by the altitudinal gradient (not by large geographical scale), is higher in the Atlantic territory (PENP) and plays a decisive role in segregating species by the identification of factors that drive the gain or loss of suitable environments. Finally, the highest PENP areas are under Alpine climate, where minimum temperatures are significantly lower than those in the lowlands. Thus, in this park, the variables that reflect the geographical and climatic conditions of high mountains (altitude, slope and minimum mean temperature of the coldest month) highly contribute to modelling the distribution of species. In this way, solar radiation could be a limiting factor only in PENP due to lower energy inputs (Moreno-Rueda & Pizarro, 2009), as shown by the difference between the reptiles from both territories for solar radiation at spring equinox.

### Differences between amphibians and reptiles

Species strongly linked to aquatic habitats showed a sudden decrease in the probability of occurrence for the lowest distance to the hydrological network values (e.g., Fig. S1). This pattern was consistent, but not exclusive among amphibians whose biology is more dependent on aquatic environments. An additional reason can support this finding: first, there was a clear correlation between distance to the hydrological network and slope (i.e., high slopes are sometimes far from the hydrological network) and second, large amphibians likely avoid areas of steep slopes and consequently maximise their probability of appearing on plains. Meanwhile, reptiles reach higher altitude ranges than amphibians, and the habitats of some reptiles are restricted to uplands (e.g., *Iberolacerta monticola* or *Zootoca vivipera*). Therefore, altitude plays a decisive role in predicting reptile distribution. Several reptile species showed a high contribution of spatial distribution of tree vegetation, indicating the sensibility of reptile species for canopy (Ferreira, Žagar & Santos, in press). For example, some species showed a preference for open woodland with an irregular discontinuous distribution of trees (*Psammodromus hispanicus, Iberolacerta monticola* and *Coronella austriaca*), whereas other species showed a preference for more uniform areas (*Lacerta schreiberi* in PENP). Fraction of canopy cover and vegetation structure in treeless areas were relevant for species such as *Zootoca vivipara* (Fig. S2), a specialist species that lives in peat bogs and stream edges covered by grasses (Hermida-Lorenzo & Lamas-Antón, 2005). Similarly, the few cases where solar radiation was relevant were mostly for reptiles (e.g., *Vipera seoanei*). These results show that factors related to solar energy at ground level are the most relevant ones for reptiles (Aragón et al., 2010).

### Mediterranean and non-Mediterranean species in both parks

We found some differences in the contribution of EGVs and the response curves according to the biogeographic affinity of species. This is consistent with previous studies that demonstrated different microhabitat selections (Ferreira, Žagar & Santos, in press) and opposing responses to disturbance (Santos & Cheylan, 2013; Ferreira, Mateus & Santos, 2016) between Mediterranean and non-Mediterranean reptiles. In this study, the biogeographic component can be confounded with the site; for this reason, species inhabiting both national parks can be illustrative of intra-specific differences in ecological niches at local scale. Some of these species were at the edge of their distribution in the Iberian Peninsula (e.g., *Lacerta schreiberi* and *Salamandra salamandra* in CNP, *Natrix maura* and *Timon lepidus* in PENP). We noted in these cases that just one or a few variables had far higher values of useful information in the models, except for *Lacerta schreiberi* (see Fig. S3, Tables S2 and S3). Thus, we could detect more easily which environmental factors are more decisive in the absence of the most optimal ecological conditions for these species in their distribution limits (CNP or PENP).

Some variables showed opposing response curves in regard to the species’ biogeographic affinity. For example, spatial distribution of tree vegetation and fraction of canopy cover show that open landscapes are preferred by most species in CNP, with a few exceptions, such as *Lacerta schreiberi* and *Salamandra salamandra*, a result that supports their non-Mediterranean habitat preferences (Pleguezuelos, Márquez & Lizana, 2002). Opposing responses in regards to the contribution to the models were observed for solar radiation at summer solstice: some non-Mediterranean species prefer shady (i.e., *Natrix natrix* and *Lacerta schreiberi* in CNP), whereas typical Mediterranean species prefer sunny spots (i.e., *Bufo calamita* and *Tarentola mauritanica* in CNP). Thus, the response to insolation and tree cover is consistent with the different needs of each species for direct sunlight and optimal thermal environment (García-París, Montori & Herrero, 2004; Salvador, 2014) that can be linked to the biogeographic affinity of species.

For species that inhabit both study areas, the maximum probability of occurrence of the most predictive variables peaked at different values in each park. Furthermore, slopes of their response curves were more accentuated in the territory out of their biogeographic region. Thus, species with non-Mediterranean affinity in the Mediterranean CNP and species with Mediterranean affinity in the Atlantic PENP seem to inhabit different realised niches with narrower EGV values.

### Final remarks

The complex information derived from the study of a large number of species may provide a global approach for studying the entire community of species and creating an integrated management process (Ferrier & Guisan, 2006). At this point, it is convenient to consider that there are different trends in each national park as well as in those species at the edge of their distribution on which factors have the most predictive ability. In this sense, this study questions the rationale for using models performed at regional or global scales (e.g., Iberian Peninsula or Europa) to establish criterions of conservation at local scales (e.g., in both national parks). Likewise, our findings are an example that ENMs performed at local scale should not be regarded as a valid framework in other areas with different environmental conditions.

Building ENMs of species at their distribution limit could provide more accurate information for conservation of herptiles in well-preserved areas. In this context, further studies on how distribution patterns respond differently depending on the degree of specialisation of the ecological niche of species (tolerance) and the distance between their ecological optimum and environmental conditions (marginality) may be useful to obtain baseline information for conservation programs (Grenouillet et al., 2011). Unfortunately, there are environmental variables not analysed in this study that could improve the models. In Mediterranean regions, some species breed in ephemeral ponds (surface water for a maximum of two months) in order to avoid competition and predation pressure (Bosch & Martínez-Solano, 2003). Our layer of the distance to the hydrological network did not contain information of the ponds with such a hydroperiod because of their unpredictable occurrence. This can explain why in CNP, amphibians which breed mainly in ephemeral ponds (i.e., *Pelodytes punctatus*, *Discoglossus galgonai* and *Bufo calamita*) have the lowest contributions of distance to the hydrological network. Soil texture is another not available environmental variable that can influence microhabitat selection (Rebello de Aguiar Junior & Mendes, 2010), particularly for species that spend much of their life buried, e.g., *Blanus cinereus* (Martín, López & Salvador, 1991) or *Pelobates cultripes* (García-París, Montori & Herrero, 2004). Future accuracy of micro-scale layers is expected to improve the predictive value of local-scale ENMs.

##  Supplemental Information

10.7717/peerj.2405/supp-1 Figure S1 Response curve of *Salamandra salamandra*from MaxEnt models The figure shows as logistic prediction changes as distance of hydrological network is varied, keeping all other environmental variables at their average sample value: (A) Response curve in Cabañeros national park; (B) Response curve in Picos de Europa national park.

10.7717/peerj.2405/supp-2 Figure S2 Response of *Zootoca vivipera* from MaxEnt models The figure shows as logistic prediction changes as vegetation structure is varied in Picos de Europa national park. This variable is reclassified into 14 categories (that reflect the land use): (1) Natural Forest; (2) Forest Plantation; (3) Agrosylvopastoral system; (4) Forest temporarily treeless; (5) Scrub; (6) Pasture and meadows; (7) Devoid of vegetation; (8) Banks; (9) Agricultural land and agricultural mosaic; (10) Artificial; (11) Wetland; (12) Water; (13) Out of range; (14) Area of human influence.

10.7717/peerj.2405/supp-3 Figure S3 Jackknife test of variable importance, from MaxEnt models, of species located in both territories (CNP and PENP) Territory at the edge of specie distribution correspond to left graphics. Saturated columns represent gain with only the one variable and less saturated columns represent gain without that variable. Variable abbreviations are detailed in Table 1.

10.7717/peerj.2405/supp-4 Table S1 Matrix of correlations Coefficients of the cross correlations between grids inputs of selected environmental variables (abbreviations are shown in Table 1).

10.7717/peerj.2405/supp-5 Table S2 Species and MaxEnt models in Cabañeros National Park It is shown the list of species of amphibians and next reptiles. For each species is added number of citations (n. cit.), its biogeographic affinity (B. affinity) that can be Mediterranean (Med.) and non-Mediterranean (non-Med.), the area under the ROC curve of test data (AUC’) and the training data (AUC”) and contribution of the environmental variables (abbreviations are shown in Table 1) to MaxEnt models.

10.7717/peerj.2405/supp-6 Table S3 Species and MaxEnt models in Picos de Europa National Park It is shown the list of species of amphibians and next reptiles. For each species is added number of citations (n.cit.), its biogeographic affinity (B. affinity) that can be Mediterranean (Med.) and non-Mediterranean (non-Med.), the area under the ROC curve of test data (AUC’) and the training data (AUC”) and contribution of the environmental variables (abbreviations are shown in Table 1) to MaxEnt models.

10.7717/peerj.2405/supp-7 Data S1 Raw data of all figures

## Additional Information and Declarations

### Data Availability

The following information was supplied regarding data availability: We used existing data produced by others; the dataset has been supplied as a Supplemental Dataset. It can also be requested from: http://www.herpetologica.es/programas/base-de-datos-herpetologica.
