# Supplementary figures and images for: Local-scale models reveal ecological niche variability in amphibian and reptile communities from two contrasting biogeographic regions
Source: PeerJ. 2016 Oct 6;4:e2405. doi: 10.7717/peerj.2405 (PMC5068418; doi:10.7717/peerj.2405)

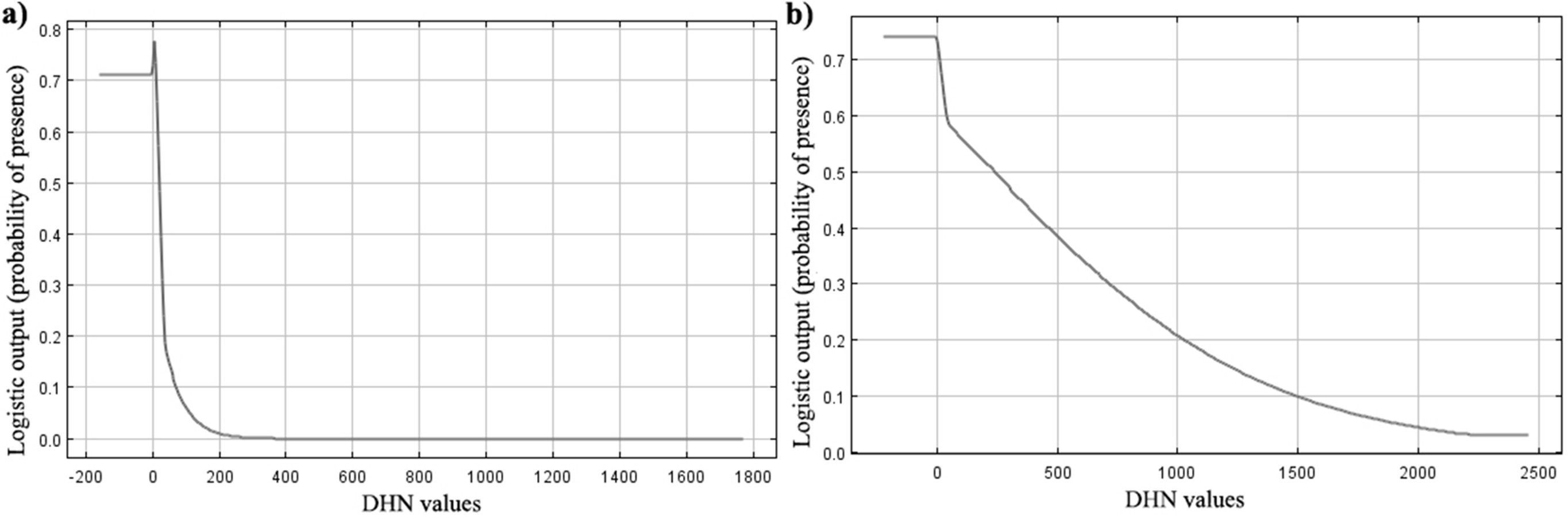

Supplement: Figure S1 — The figure shows as logistic prediction changes as distance of hydrological network is varied, keeping all other environmental variables at their average sample value: (A) Response curve in Cabañeros national park; (B) Response curve in Picos de Europa national park. [file peerj-04-2405-s001.png]

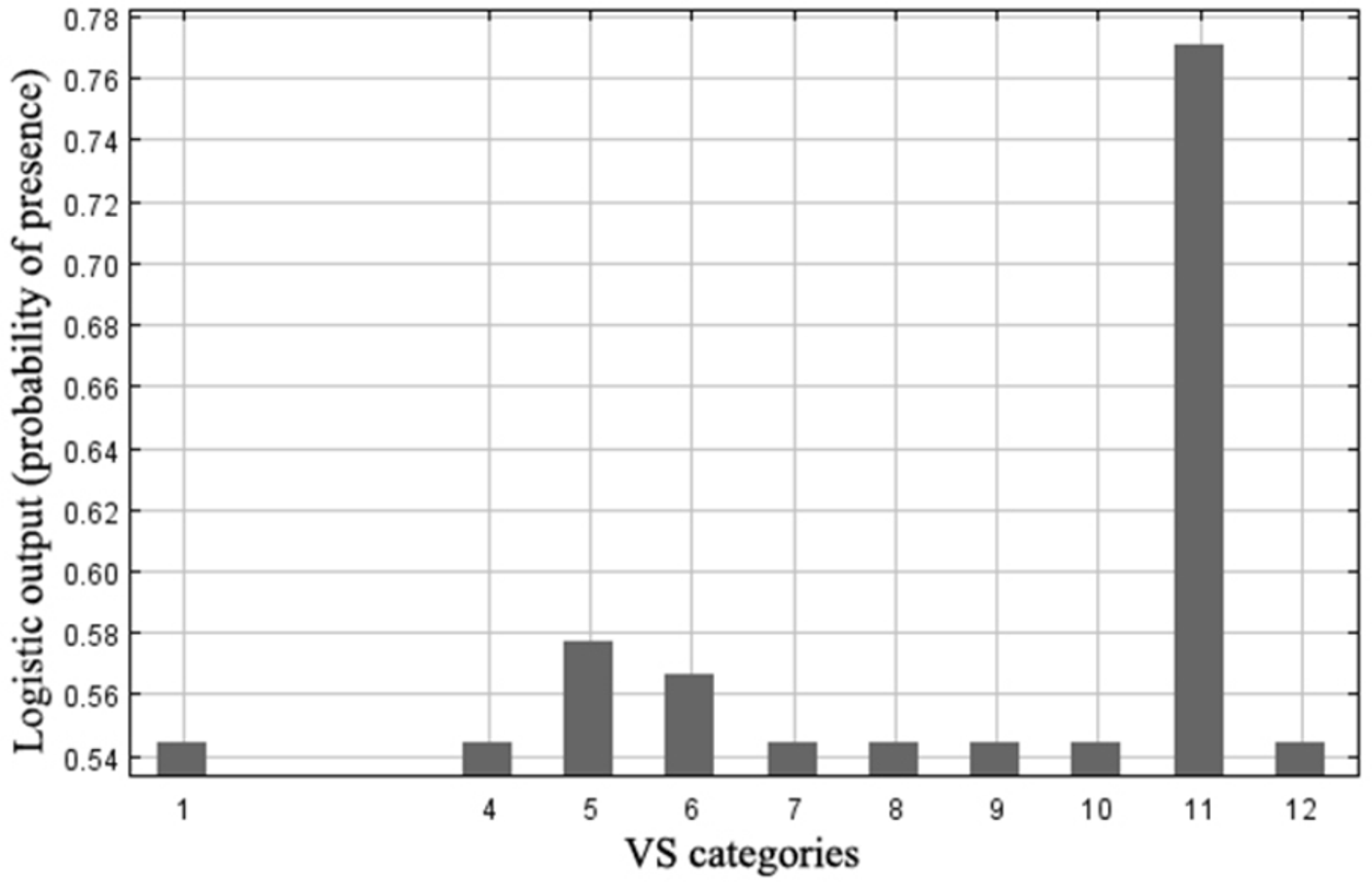

Supplement: Figure S2 — The figure shows as logistic prediction changes as vegetation structure is varied in Picos de Europa national park. This variable is reclassified into 14 categories (that reflect the land use): (1) Natural Forest; (2) Forest Plantation; (3) Agrosylvopastoral system; (4) Forest temporarily treeless; (5) Scrub; (6) Pasture and meadows; (7) Devoid of vegetation; (8) Banks; (9) Agricultural land and agricultural mosaic; (10) Artificial; (11) Wetland; (12) Water; (13) Out of range; (14) Area of human influence. [file peerj-04-2405-s002.png]

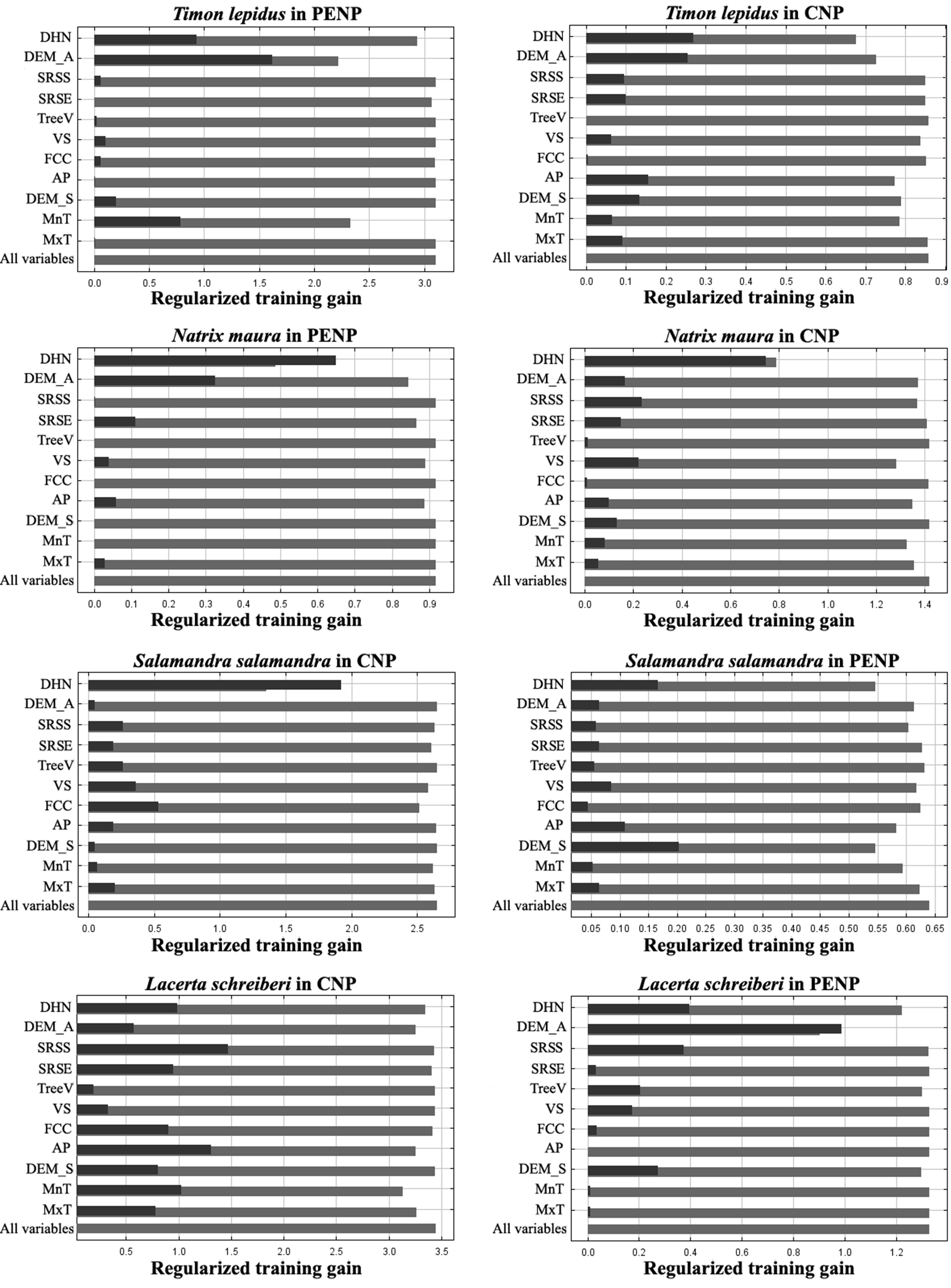

Supplement: Figure S3 — Territory at the edge of specie distribution correspond to left graphics. Saturated columns represent gain with only the one variable and less saturated columns represent gain without that variable. Variable abbreviations are detailed in Table 1. [file peerj-04-2405-s003.png]

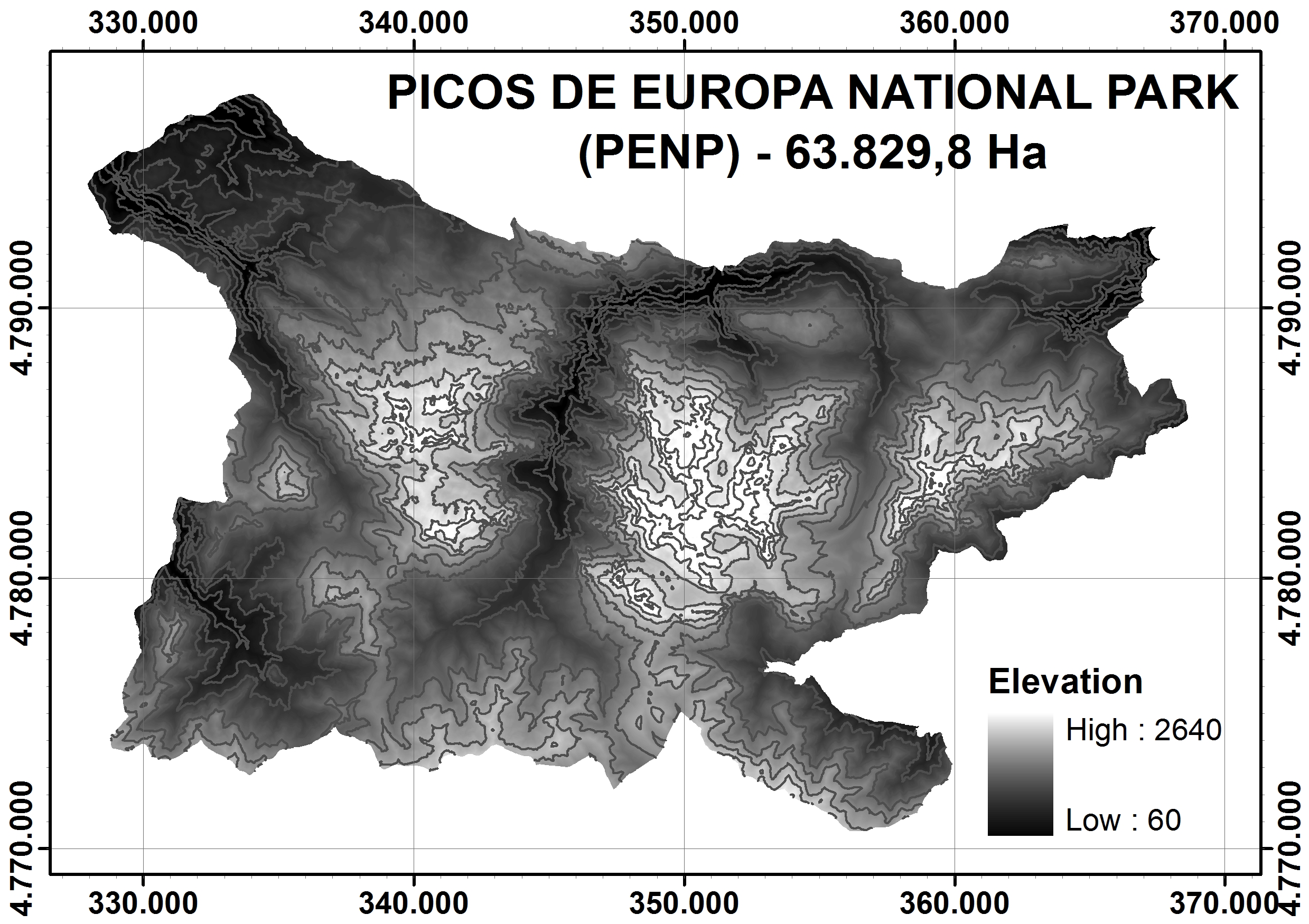

Supplement: Data S1 [file peerj-04-2405-s007.zip › Raw_Data_Fig1 (1).jpg]

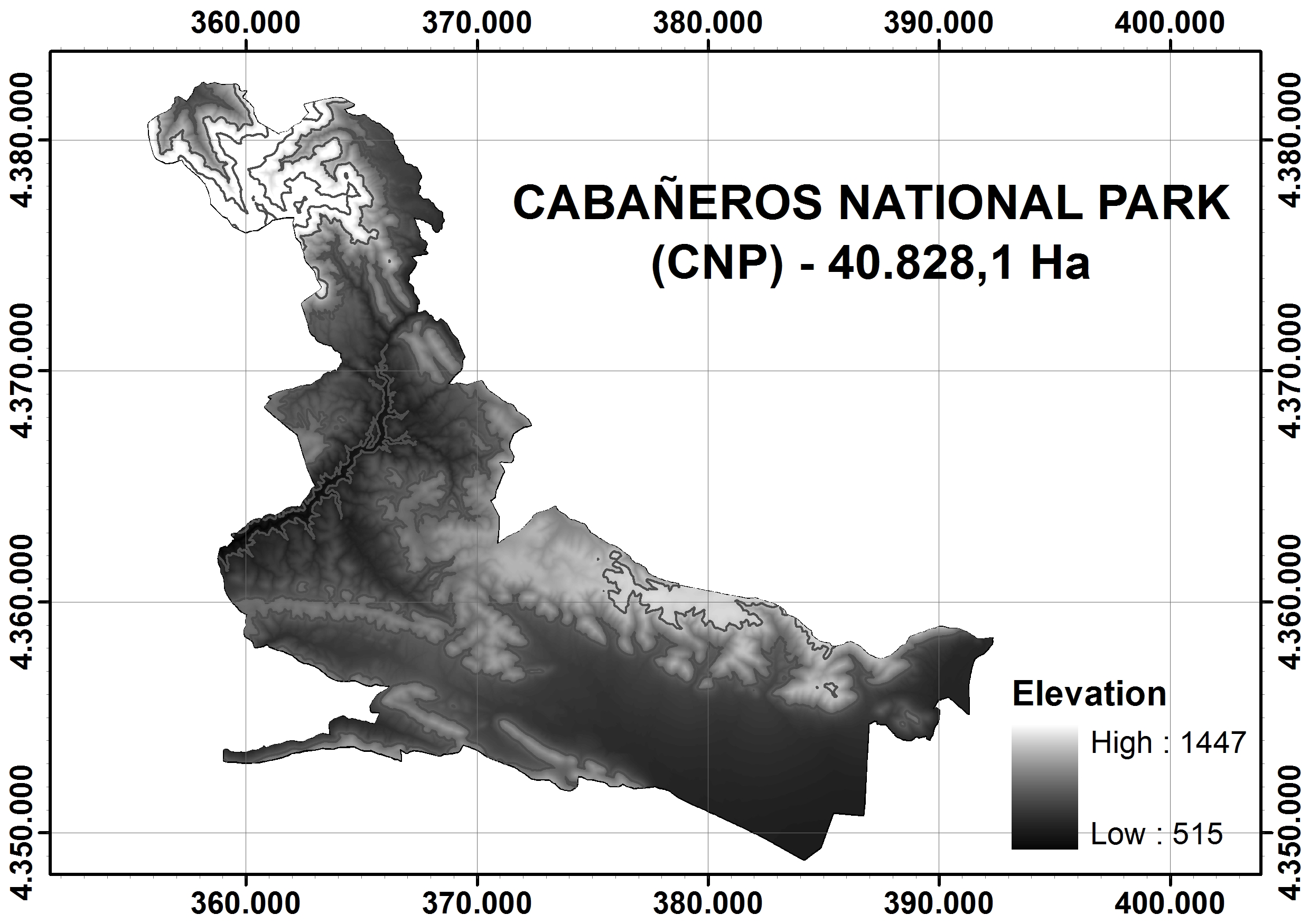

Supplement: Data S1 [file peerj-04-2405-s007.zip › Raw_Data_Fig1 (2).jpg]

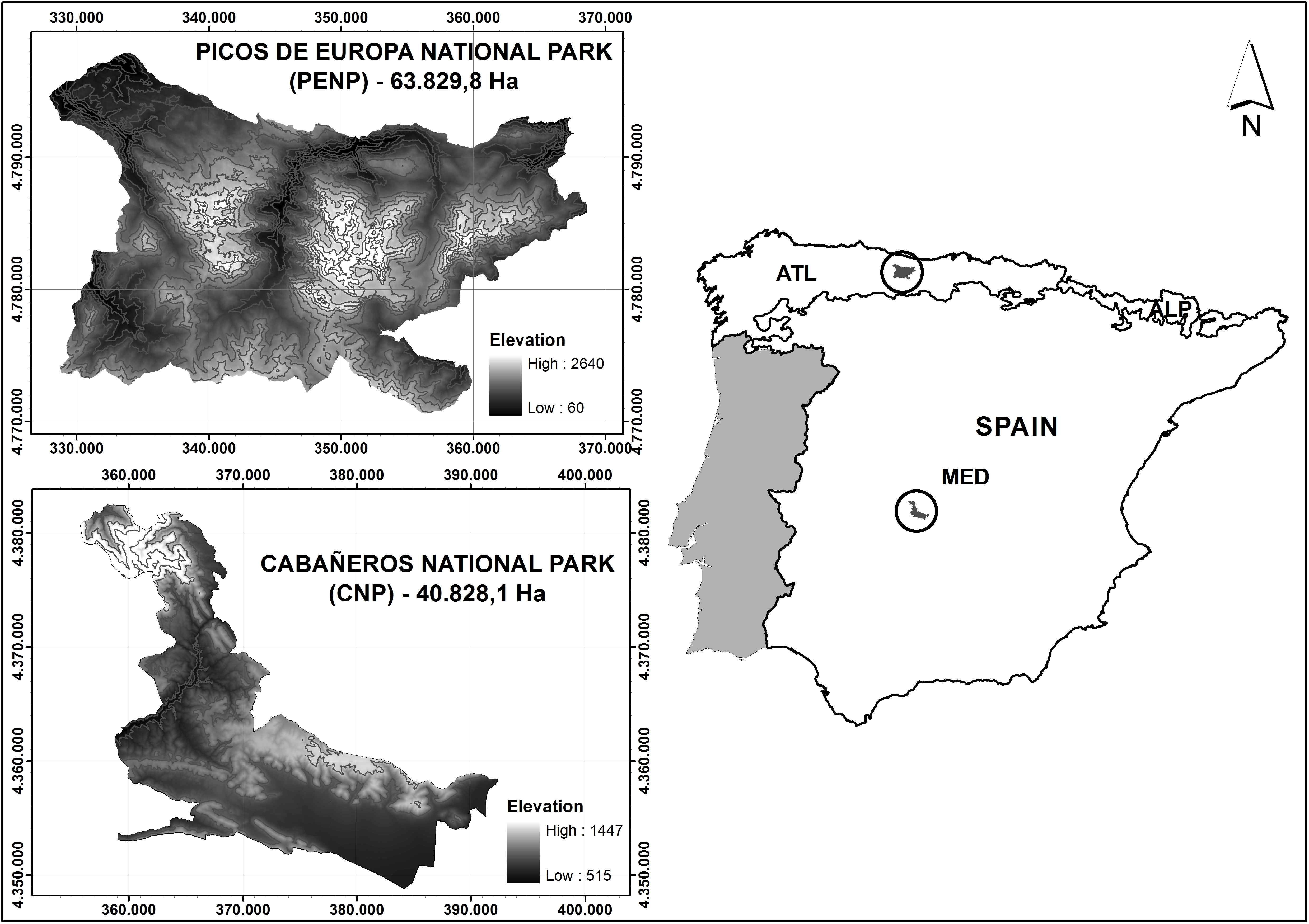

Supplement: Data S1 [file peerj-04-2405-s007.zip › Raw_Data_Fig1 (3).jpg]

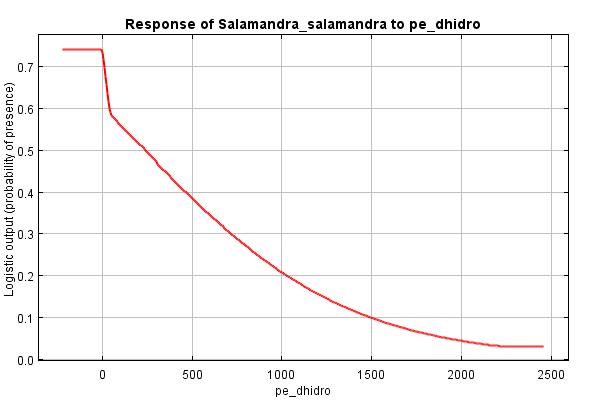

Supplement: Data S1 [file peerj-04-2405-s007.zip › Raw_Data_MaxEnt_output_Fig5 (1).png]

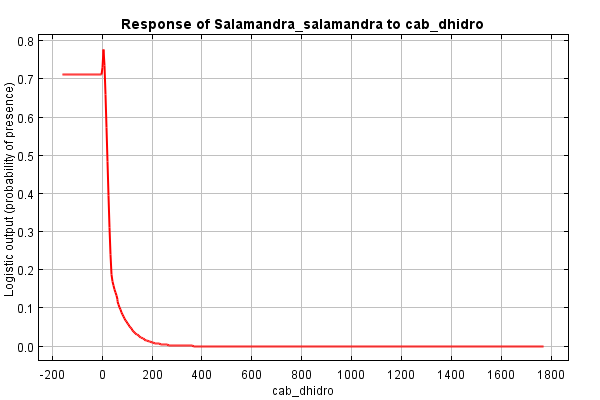

Supplement: Data S1 [file peerj-04-2405-s007.zip › Raw_Data_MaxEnt_output_Fig5 (2).png]

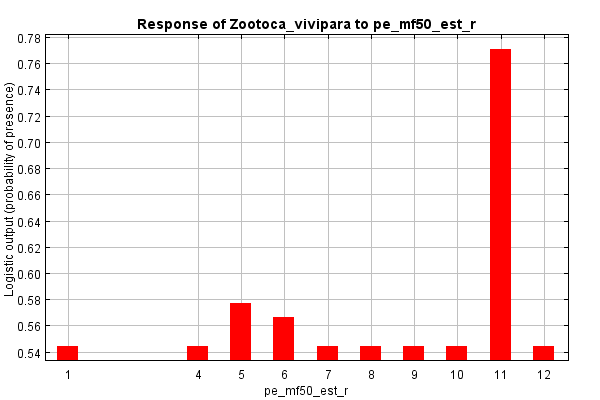

Supplement: Data S1 [file peerj-04-2405-s007.zip › Raw_Data_MaxEnt_output_Fig6.png]

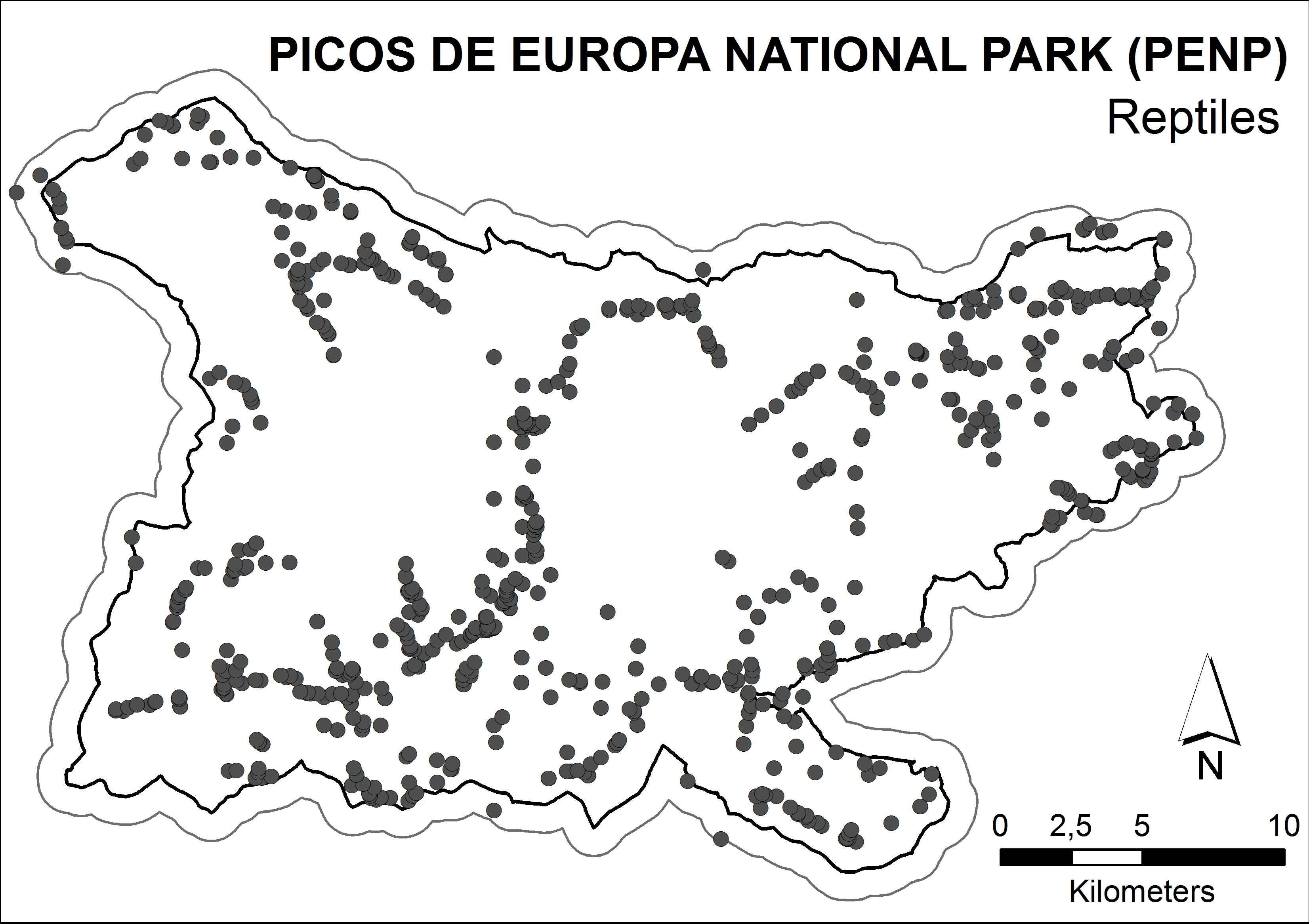

Supplement: Data S1 [file peerj-04-2405-s007.zip › Raw_Data_SI_Fig_S1 (1).jpg]

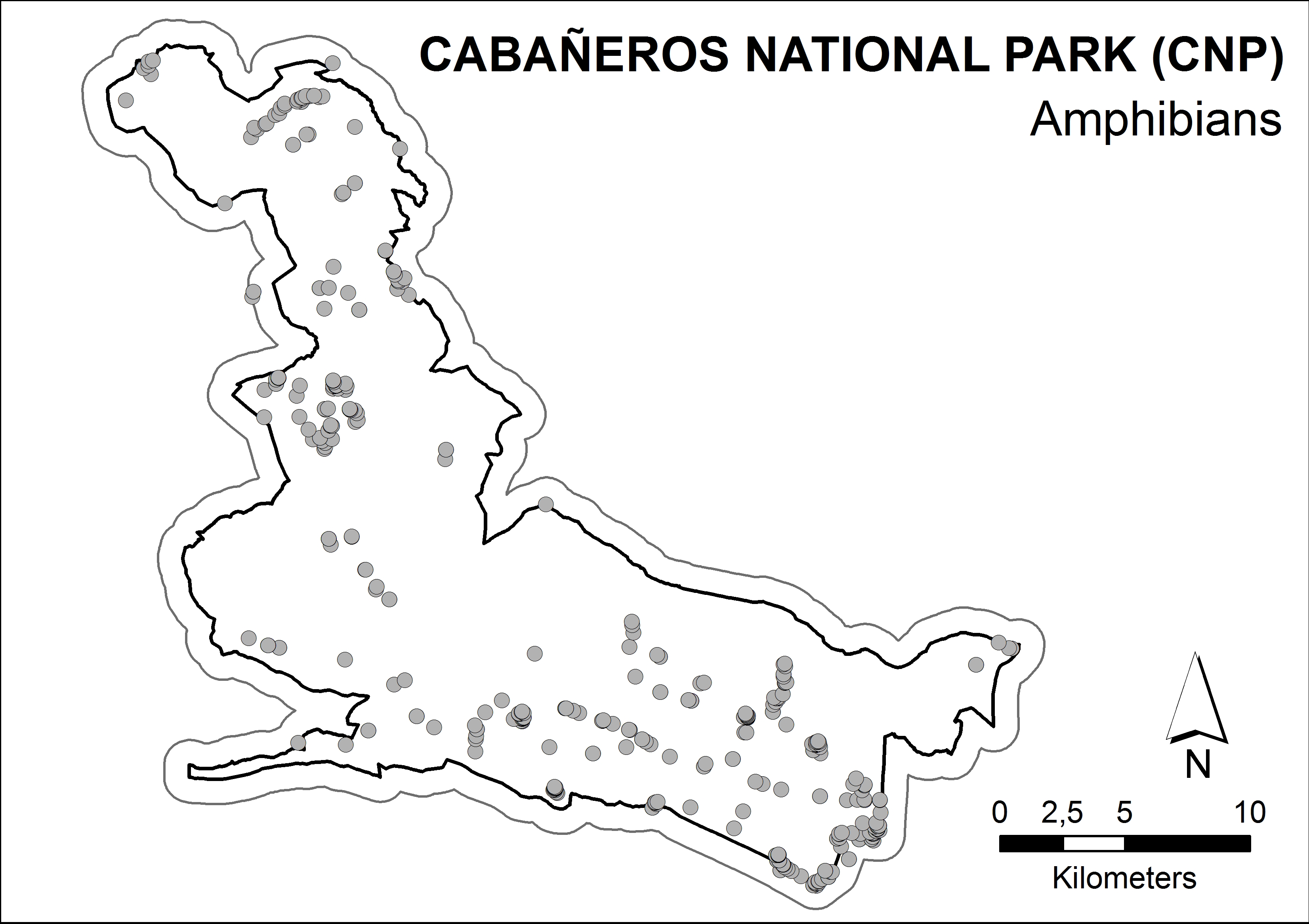

Supplement: Data S1 [file peerj-04-2405-s007.zip › Raw_Data_SI_Fig_S1 (2).jpg]

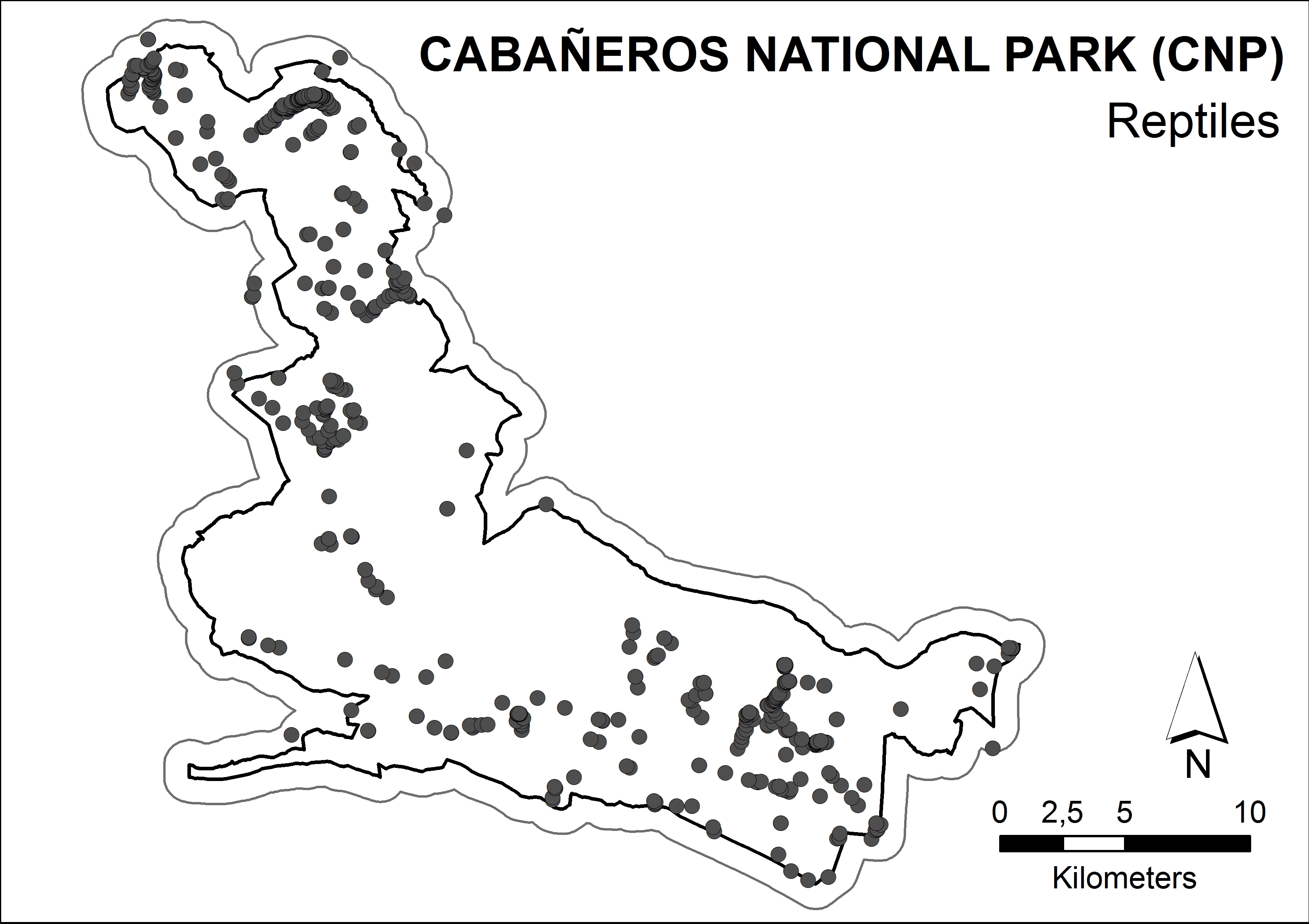

Supplement: Data S1 [file peerj-04-2405-s007.zip › Raw_Data_SI_Fig_S1 (3).jpg]

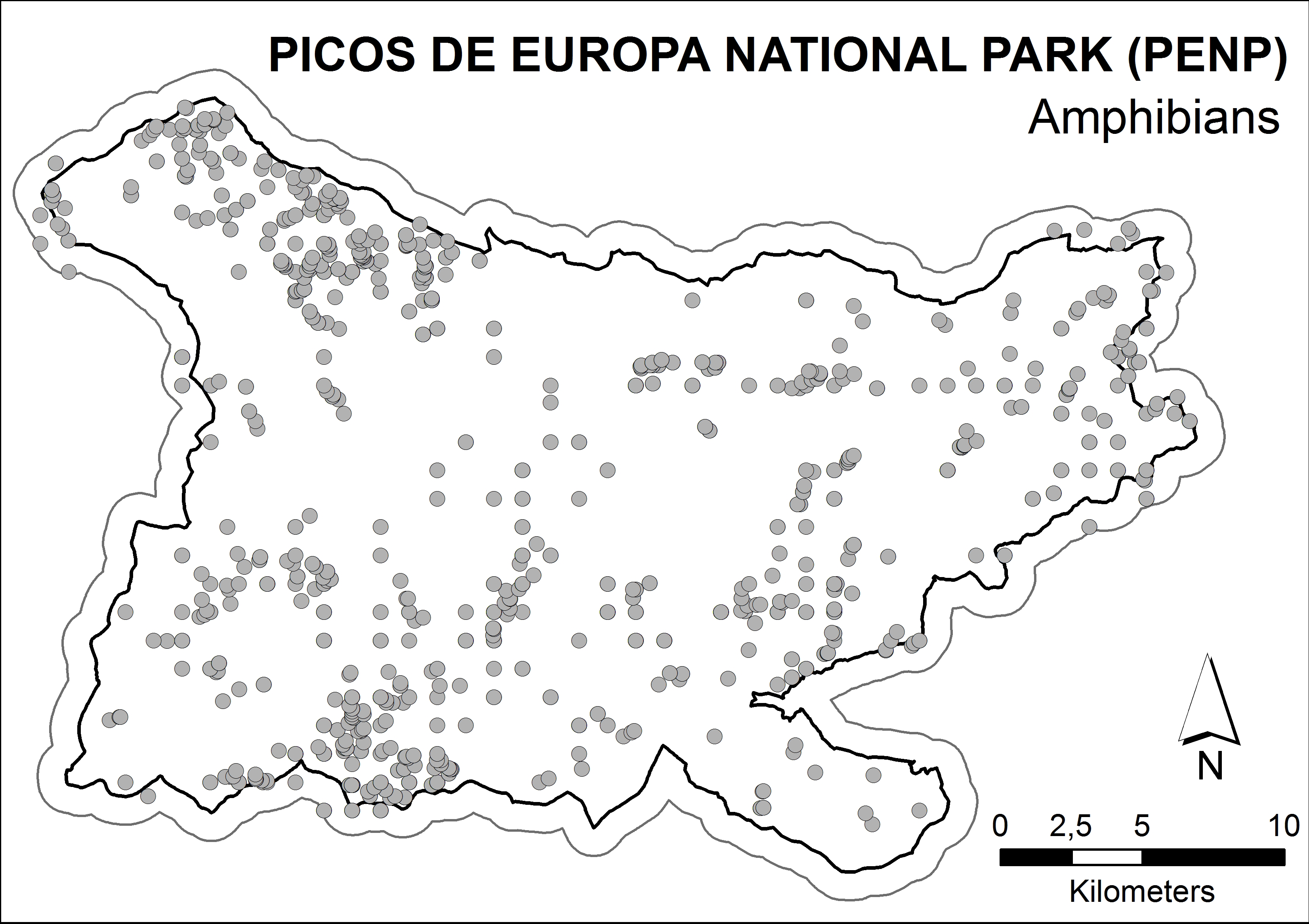

Supplement: Data S1 [file peerj-04-2405-s007.zip › Raw_Data_SI_Fig_S1 (4).jpg]

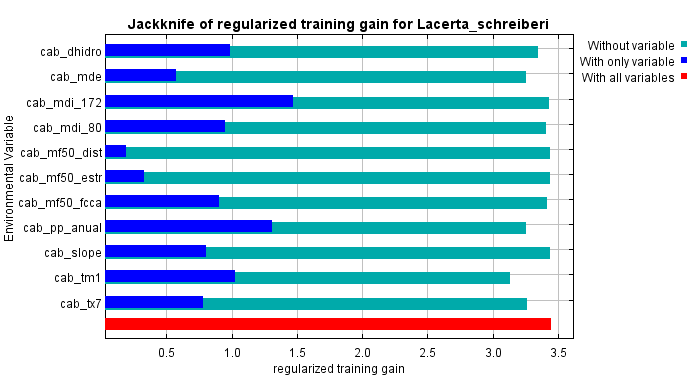

Supplement: Data S1 [file peerj-04-2405-s007.zip › Raw_Data_SI_MaxEnt_output_Fig_S2 (1).png]

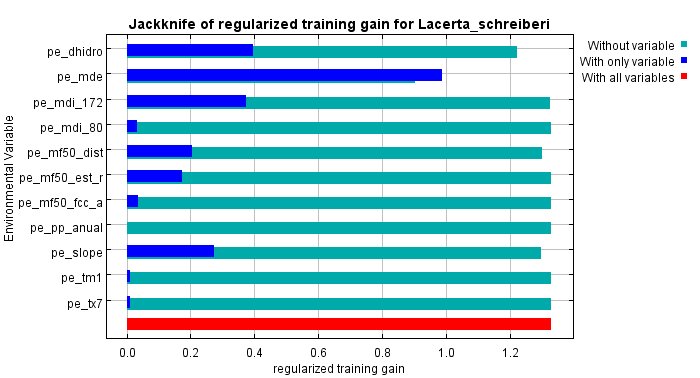

Supplement: Data S1 [file peerj-04-2405-s007.zip › Raw_Data_SI_MaxEnt_output_Fig_S2 (2).png]

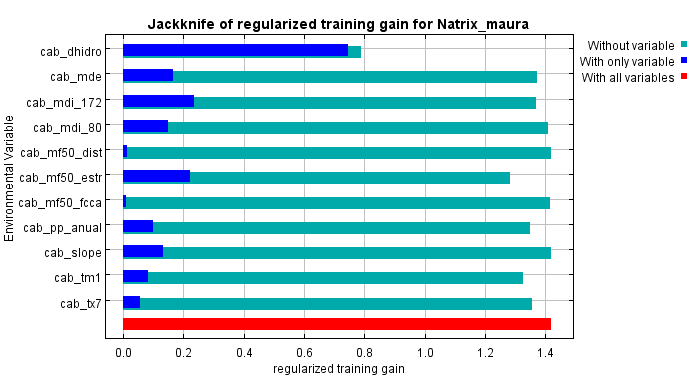

Supplement: Data S1 [file peerj-04-2405-s007.zip › Raw_Data_SI_MaxEnt_output_Fig_S2 (3).png]

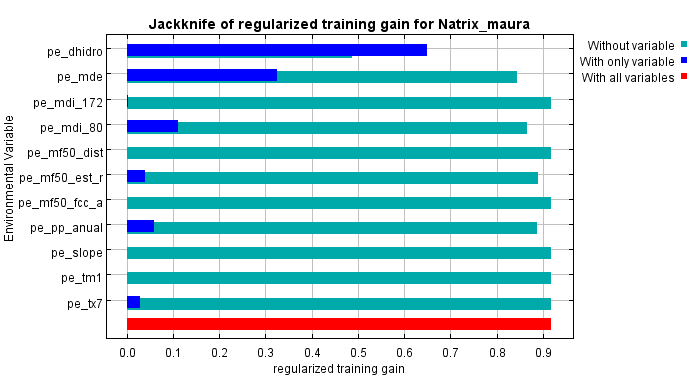

Supplement: Data S1 [file peerj-04-2405-s007.zip › Raw_Data_SI_MaxEnt_output_Fig_S2 (4).png]

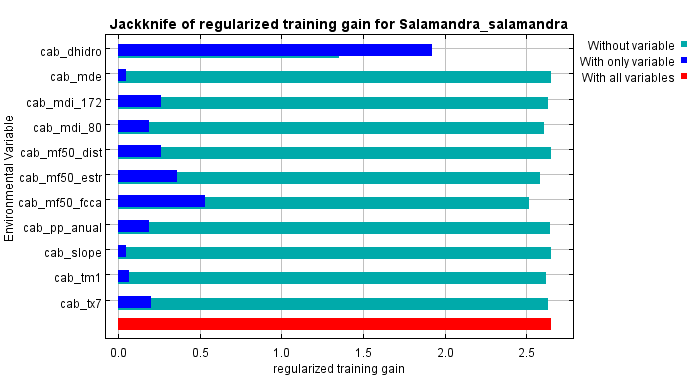

Supplement: Data S1 [file peerj-04-2405-s007.zip › Raw_Data_SI_MaxEnt_output_Fig_S2 (5).png]

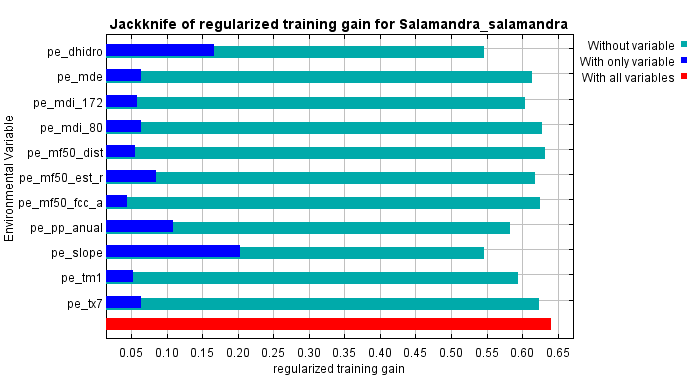

Supplement: Data S1 [file peerj-04-2405-s007.zip › Raw_Data_SI_MaxEnt_output_Fig_S2 (6).png]

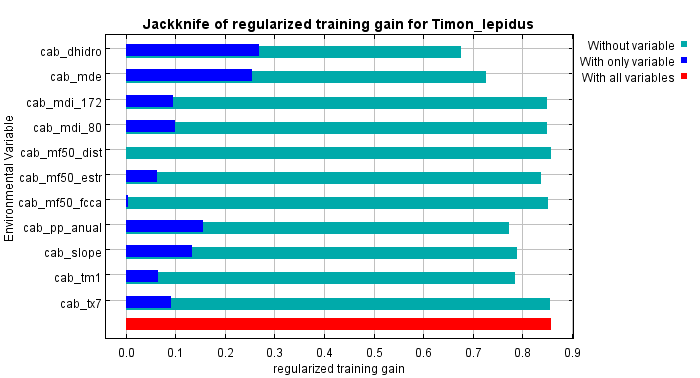

Supplement: Data S1 [file peerj-04-2405-s007.zip › Raw_Data_SI_MaxEnt_output_Fig_S2 (7).png]

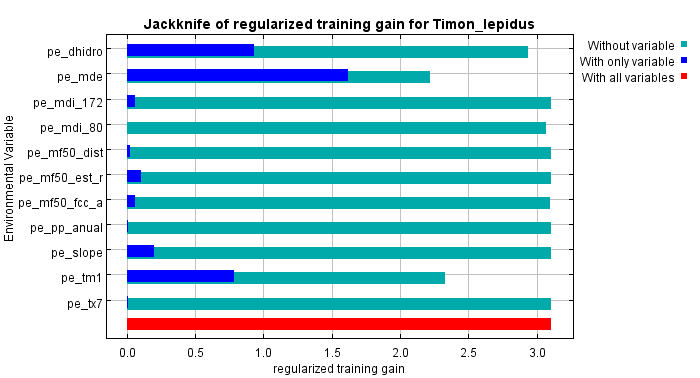

Supplement: Data S1 [file peerj-04-2405-s007.zip › Raw_Data_SI_MaxEnt_output_Fig_S2 (8).png]
