# Supplementary material for: Local-scale models reveal ecological niche variability in amphibian and reptile communities from two contrasting biogeographic regions
Source: PeerJ. 2016 Oct 6;4:e2405. doi: 10.7717/peerj.2405 (PMC5068418; doi:10.7717/peerj.2405)
Supplement: Table S1 — Coefficients of the cross correlations between grids inputs of selected environmental variables (abbreviations are shown in Table 1). [file peerj-04-2405-s004.docx]

|  |  | **DHN** | **DEM_A** | **DEM_S** | **SRSS** | **SRSE** | **TreeV** | **VS** | **FCC** | **AP** | **MnT** | **MxT** |
| --- | --- | --- | --- | --- | --- | --- | --- | --- | --- | --- | --- | --- |
| CABAÑEROS NATIONAL PARK | **DHN** | - | 0,07 | 0,12 | 0,05 | 0,12 | 0,08 | 0,17 | 0,10 | 0,24 | 0,21 | 0,16 |
|  | **DEM_A** | 0,07 | - | 0,42 | 0,09 | 0,31 | 0,20 | 0,35 | 0,27 | 0,06 | 0,12 | 0,08 |
|  | **DEM_S** | 0,12 | 0,42 | - | 0,20 | 0,72 | 0,20 | 0,38 | 0,27 | 0,25 | 0,08 | 0,31 |
|  | **SRSS** | 0,05 | 0,09 | 0,20 | - | 0,62 | 0,10 | 0,13 | 0,12 | 0,72 | 0,04 | 0,09 |
|  | **SRSE** | 0,12 | 0,31 | 0,72 | 0,62 | - | 0,18 | 0,29 | 0,24 | 0,20 | 0,12 | 0,25 |
|  | **TreeV** | 0,08 | 0,20 | 0,20 | 0,10 | 0,19 | - | 0,73 | 0,65 | 0,23 | 0,34 | 0,18 |
|  | **VS** | 0,17 | 0,35 | 0,38 | 0,13 | 0,29 | 0,73 | - | 0,69 | 0,14 | 0,01 | 0,10 |
|  | **FCC** | 0,10 | 0,27 | 0,27 | 0,12 | 0,24 | 0,65 | 0,69 | - | 0,12 | 0,07 | 0,04 |
|  | **AP** | 0,24 | 0,06 | 0,25 | 0,72 | 0,20 | 0,23 | 0,14 | 0,12 | - | 0,36 | 0,45 |
|  | **MnT** | 0,21 | 0,12 | 0,08 | 0,04 | 0,12 | 0,34 | 0,01 | 0,07 | 0,36 | - | 0,42 |
|  | **MxT** | 0,16 | 0,08 | 0,31 | 0,09 | 0,25 | 0,18 | 0,10 | 0,04 | 0,45 | 0,42 | - |
| PICOS DE EUROPA NATIONAL PARK | **DHN** | - | 0,41 | 0,60 | 0,06 | 0,04 | 0,12 | 0,24 | 0,21 | 0,09 | 0,08 | 0,10 |
|  | **DEM_A** | 0,41 | - | 0,01 | 0,06 | 0,09 | 0,33 | 0,38 | 0,32 | 0,02 | 0,44 | 0,12 |
|  | **DEM_S** | 0,60 | 0,01 | - | 0,24 | 0,66 | 0,23 | 0,60 | 0,69 | 0,08 | 0,07 | 0,09 |
|  | **SRSS** | 0,06 | 0,06 | 0,24 | - | 0,68 | 0,08 | 0,13 | 0,15 | 0,10 | 0,06 | 0,06 |
|  | **SRSE** | 0,04 | 0,09 | 0,66 | 0,68 | - | 0,02 | 0,01 | 0,02 | 0,10 | 0,08 | 0,09 |
|  | **TreeV** | 0,12 | 0,33 | 0,23 | 0,08 | 0,02 | - | 0,56 | 0,49 | 0,12 | 0,05 | 0,09 |
|  | **VS** | 0,24 | 0,38 | 0,60 | 0,13 | 0,01 | 0,56 | - | 0,74 | 0,17 | 0,12 | 0,21 |
|  | **FCC** | 0,21 | 0,32 | 0,69 | 0,15 | 0,02 | 0,49 | 0,74 | - | 0,15 | 0,15 | 0,17 |
|  | **AP** | 0,09 | 0,02 | 0,08 | 0,10 | 0,10 | 0,12 | 0,17 | 0,15 | - | 0,40 | 0,69 |
|  | **MnT** | 0,08 | 0,44 | 0,07 | 0,06 | 0,08 | 0,05 | 0,12 | 0,15 | 0,40 | - | 0,67 |
|  | **MxT** | 0,10 | 0,12 | 0,09 | 0,06 | 0,09 | 0,09 | 0,21 | 0,17 | 0,69 | 0,67 | - |
