# Supplementary material for: Local-scale models reveal ecological niche variability in amphibian and reptile communities from two contrasting biogeographic regions
Source: PeerJ. 2016 Oct 6;4:e2405. doi: 10.7717/peerj.2405 (PMC5068418; doi:10.7717/peerj.2405)
Supplement: Table S2 — It is shown the list of species of amphibians and next reptiles. For each species is added number of citations (n. cit.), its biogeographic affinity (B. affinity) that can be Mediterranean (Med.) and non-Mediterranean (non-Med.), the area under the ROC curve of test data (AUC’) and the training data (AUC”) and contribution of the environmental variables (abbreviations are shown in Table 1) to MaxEnt models. [file peerj-04-2405-s005.docx]

| **Specie** | **n. cit.** | **B. affinity** | **AUC'** | **AUC''** | **DHN** | **DEM_A** | **DEM_S** | **SRSE** | **SRSS** | **TreeV** | **VS** | **FCC** | **AP** | **MnT** | **MxT** |
| --- | --- | --- | --- | --- | --- | --- | --- | --- | --- | --- | --- | --- | --- | --- | --- |
| *Alytes cisternasii* | 11 | Med. | 0,97 | 0,95 | 39,8 | 27,3 | 0,0 | 0,0 | 0,0 | 0,0 | 20,6 | 12,3 | 0,0 | 0,0 | 0,0 |
| *Bufo calamita* | 43 | Med. | 0,95 | 0,93 | 7,5 | 0,7 | 46,5 | 0,2 | 21,9 | 2,6 | 1,1 | 0,1 | 14,5 | 1,9 | 3,1 |
| *Bufo spinosus* | 55 | Med. | 0,88 | 0,93 | 43,0 | 3,4 | 4,4 | 0,1 | 6,5 | 0,0 | 3,1 | 4,7 | 4,0 | 24,0 | 6,9 |
| *Discoglossus galganoi* | 11 | Med. | 0,87 | 0,90 | 6,2 | 11,6 | 57,0 | 0,0 | 0,0 | 0,0 | 24,4 | 0,0 | 0,0 | 0,0 | 0,8 |
| *Hyla molleri* | 51 | Med. | 0,92 | 0,94 | 37,8 | 11,8 | 28,7 | 0,2 | 1,9 | 0,0 | 6,4 | 0,1 | 8,5 | 0,7 | 3,9 |
| *Lissotriton boscai* | 63 | Med. | 0,92 | 0,95 | 78,0 | 2,5 | 3,1 | 2,1 | 1,7 | 0,0 | 2,5 | 1,1 | 3,8 | 4,7 | 0,4 |
| *Pelobates cultripes* | 45 | Med. | 0,98 | 0,97 | 12,3 | 6,3 | 19,7 | 0,7 | 3,0 | 0,0 | 37,7 | 1,7 | 14,3 | 0,6 | 3,5 |
| *Pelodytes punctatus* | 20 | Med. | 0,9 | 0,98 | 3,1 | 2,1 | 24,5 | 0,3 | 2,8 | 3,6 | 41,7 | 4,7 | 12,0 | 0,2 | 5,0 |
| *Pelophylax perezi* | 152 | Med. | 0,9 | 0,93 | 58,9 | 17,4 | 5,9 | 2,1 | 1,7 | 0,0 | 0,5 | 1,5 | 6,8 | 2,8 | 2,4 |
| *Pleurodeles waltl* | 44 | Med. | 0,93 | 0,96 | 44,8 | 1,5 | 20,5 | 1,3 | 1,0 | 0,1 | 4,7 | 3,7 | 8,4 | 8,3 | 5,8 |
| *Salamandra salamandra* | 20 | non-Med. | 0,98 | 0,98 | 71,3 | 0,0 | 0,1 | 1,9 | 1,6 | 0,0 | 4,4 | 15,7 | 1,9 | 1,3 | 1,8 |
| *Triturus pygmaeus* | 14 | Med. | 0,75 | 0,94 | 21,3 | 7,2 | 27,5 | 0,0 | 0,0 | 0,0 | 24,7 | 0,0 | 1,7 | 0,0 | 17,6 |
| *Blanus cinereus* | 22 | Med. | 0,69 | 0,95 | 46,8 | 15,6 | 5,4 | 1,1 | 5,7 | 0,0 | 5,0 | 0,0 | 13,9 | 4,6 | 2,0 |
| *Chalcides bedriagai* | 5 | Med. | 0,41 | 0,90 | 11,2 | 0,0 | 0,0 | 0,0 | 14,5 | 0,0 | 26,3 | 0,0 | 48,0 | 0,0 | 0,0 |
| *Chalcides striatus* | 8 | Med. | 0,34 | 0,91 | 32,2 | 0,0 | 18,8 | 0,0 | 0,0 | 4,5 | 44,4 | 0,0 | 0,0 | 0,0 | 0,0 |
| *Emys orbicularis* | 31 | Med. | 0,94 | 0,96 | 57,9 | 7,4 | 7,9 | 0,3 | 1,4 | 3,2 | 5,7 | 0,4 | 1,4 | 3,2 | 11,2 |
| *Hemorrhois hippocrepis* | 11 | Med. | 0,72 | 0,85 | 27,5 | 1,8 | 0,0 | 0,0 | 0,0 | 8,8 | 2,3 | 0,0 | 3,0 | 55,7 | 0,9 |
| *Lacerta schreiberi* | 94 | non-Med. | 0,99 | 0,99 | 19,4 | 13,5 | 0,1 | 1,2 | 28,9 | 0,0 | 0,4 | 1,9 | 10,0 | 9,6 | 15,1 |
| *Macroprotodon brevis* | 4 | Med. | 0,47 | 0,84 | 0,0 | 0,0 | 67,1 | 0,0 | 3,1 | 0,0 | 9,2 | 20,5 | 0,0 | 0,0 | 0,0 |
| *Malpolon monspessulanus* | 31 | Med. | 0,79 | 0,89 | 16,8 | 19,4 | 18,5 | 0,0 | 0,3 | 0,0 | 6,6 | 0,9 | 12,5 | 20,8 | 4,1 |
| *Mauremys leprosa* | 46 | Med. | 0,89 | 0,94 | 75,5 | 3,2 | 0,7 | 0,6 | 0,3 | 0,0 | 2,8 | 1,9 | 4,6 | 6,4 | 4,0 |
| *Natrix maura* | 46 | Med. | 0,87 | 0,94 | 57,1 | 0,7 | 0,0 | 2,6 | 4,0 | 0,0 | 20,7 | 0,2 | 5,9 | 5,5 | 3,2 |
| *Natrix natrix* | 21 | non-Med. | 0,99 | 0,99 | 45,7 | 1,9 | 5,3 | 0,1 | 23,8 | 0,0 | 1,4 | 2,6 | 4,0 | 12,0 | 3,2 |
| *Podarcis virescens* | 55 | Med. | 0,8 | 0,89 | 63,3 | 1,3 | 1,8 | 0,8 | 0,4 | 0,0 | 10,5 | 2,6 | 8,7 | 9,1 | 1,4 |
| *Psammodromus algirus* | 72 | Med. | 0,83 | 0,91 | 36,5 | 17,0 | 0,0 | 0,6 | 1,4 | 0,5 | 4,8 | 1,2 | 15,5 | 8,4 | 14,2 |
| *Psammodromus hispanicus* | 12 | Med. | 0,82 | 0,81 | 0,0 | 1,9 | 68,7 | 0,3 | 0,0 | 20,0 | 3,7 | 0,0 | 0,1 | 5,3 | 0,0 |
| *Rhinechis scalaris* | 7 | Med. | 0,81 | 0,88 | 80,3 | 16,4 | 0,0 | 0,0 | 0,0 | 0,0 | 3,2 | 0,0 | 0,0 | 0,2 | 0,0 |
| *Tarentola mauritanica* | 15 | Med. | 0,58 | 0,93 | 7,0 | 3,7 | 37,9 | 1,1 | 17,8 | 0,7 | 8,2 | 0,0 | 11,4 | 10,8 | 1,4 |
| *Timon lepidus* | 92 | Med. | 0,78 | 0,88 | 27,0 | 25,6 | 6,2 | 1,1 | 6,8 | 0,0 | 2,8 | 0,1 | 14,4 | 10,8 | 5,3 |
| *Vipera latastei* | 5 | Med. | 0,97 | 0,95 | 39,6 | 0,0 | 0,0 | 0,0 | 0,0 | 0,0 | 5,0 | 0,0 | 53,3 | 0,1 | 2,0 |
