# Supplementary material for: Local-scale models reveal ecological niche variability in amphibian and reptile communities from two contrasting biogeographic regions
Source: PeerJ. 2016 Oct 6;4:e2405. doi: 10.7717/peerj.2405 (PMC5068418; doi:10.7717/peerj.2405)
Supplement: Table S3 — It is shown the list of species of amphibians and next reptiles. For each species is added number of citations (n.cit.), its biogeographic affinity (B. affinity) that can be Mediterranean (Med.) and non-Mediterranean (non-Med.), the area under the ROC curve of test data (AUC’) and the training data (AUC”) and contribution of the environmental variables (abbreviations are shown in Table 1) to MaxEnt models. [file peerj-04-2405-s006.docx]

| **Specie** | **n. cit.** | **B. affinity** | **AUC'** | **AUC''** | **DHN** | **DEM_A** | **DEM_S** | **SRSE** | **SRSS** | **TreeV** | **VS** | **FCC** | **AP** | **MnT** | **MxT** |
| --- | --- | --- | --- | --- | --- | --- | --- | --- | --- | --- | --- | --- | --- | --- | --- |
| *Alytes obstetricans* | 255 | non-Med. | 0,72 | 0,80 | 28,5 | 6,7 | 38,3 | 1,6 | 1,5 | 1,1 | 5,9 | 0,8 | 5,5 | 6,3 | 3,7 |
| *Bufo spinosus* | 161 | Med. | 0,81 | 0,85 | 34,9 | 13,1 | 23,8 | 1,5 | 4,2 | 0,6 | 2,9 | 1,2 | 13,5 | 4,3 | 0,0 |
| *Chioglossa lusitanica* | 12 | non-Med. | 0,99 | 0,98 | 0,0 | 69,3 | 0,1 | 0,0 | 0,3 | 0,8 | 0,5 | 7,1 | 0,0 | 21,8 | 0,1 |
| *Lissotriton helveticus* | 165 | non-Med. | 0,83 | 0,89 | 11,0 | 10,0 | 40,7 | 1,3 | 1,3 | 0,0 | 0,9 | 1,2 | 10,5 | 19,3 | 3,8 |
| *Mesotriton alpestris* | 133 | non-Med. | 0,83 | 0,91 | 20,2 | 5,3 | 28,4 | 2,8 | 2,2 | 8,5 | 2,7 | 3,5 | 12,8 | 9,0 | 4,5 |
| *Rana iberica* | 6 | non-Med. | 0,98 | 0,99 | 37,8 | 0,4 | 1,3 | 0,0 | 0,0 | 0,1 | 0,2 | 0,0 | 0,4 | 59,8 | 0,0 |
| *Rana temporaria* | 226 | non-Med. | 0,75 | 0,84 | 15,3 | 7,4 | 52,6 | 3,8 | 2,5 | 0,0 | 0,7 | 0,6 | 7,4 | 4,4 | 5,3 |
| *Salamandra salamandra* | 145 | non-Med. | 0,7 | 0,85 | 20,2 | 4,1 | 31,4 | 3,8 | 4,7 | 6,6 | 4,4 | 0,7 | 16,0 | 6,0 | 2,1 |
| *Triturus marmoratus* | 5 | non-Med. | 0,87 | 0,99 | 0,0 | 17,3 | 3,0 | 0,0 | 0,0 | 0,0 | 21,8 | 16,2 | 0,1 | 41,6 | 0,0 |
| *Anguis fragilis* | 72 | non-Med. | 0,83 | 0,89 | 8,5 | 44,3 | 15,3 | 10,4 | 1,4 | 0,6 | 7,8 | 0,0 | 1,7 | 4,3 | 5,8 |
| *Coronella austriaca* | 39 | non-Med. | 0,87 | 0,93 | 24,8 | 26,0 | 11,7 | 5,5 | 1,1 | 11,2 | 0,7 | 0,7 | 0,9 | 16,6 | 0,8 |
| *Iberolacerta monticola* | 76 | non-Med. | 0,83 | 0,89 | 10,8 | 10,2 | 34,4 | 0,3 | 3,3 | 14,2 | 2,3 | 3,3 | 3,9 | 8,1 | 9,2 |
| *Lacerta bilineata* | 63 | non-Med. | 0,94 | 0,95 | 22,5 | 25,6 | 11,8 | 3,3 | 0,4 | 0,3 | 9,3 | 2,3 | 0,7 | 22,1 | 1,8 |
| *Lacerta schreiberi* | 10 | non-Med. | 0,88 | 0,93 | 10,5 | 59,3 | 3,8 | 0,0 | 16,7 | 9,6 | 0,0 | 0,0 | 0,0 | 0,0 | 0,0 |
| *Natrix maura* | 6 | Med. | 0,62 | 0,91 | 63,9 | 17,3 | 0,0 | 10,4 | 0,0 | 0,0 | 3,8 | 0,0 | 2,3 | 0,0 | 2,2 |
| *Natrix natrix* | 21 | non-Med. | 0,97 | 0,95 | 59,0 | 8,0 | 1,0 | 0,4 | 2,2 | 0,1 | 9,3 | 0,0 | 0,0 | 18,8 | 1,3 |
| *Podarcis guadarramae subsp. lusitanicus* | 26 | non-Med. | 0,97 | 0,99 | 3,5 | 61,1 | 0,9 | 9,7 | 0,7 | 0,3 | 4,3 | 7,4 | 0,0 | 1,6 | 10,4 |
| *Podarcis muralis* | 330 | non-Med. | 0,83 | 0,84 | 11,1 | 32,5 | 19,3 | 11,1 | 5,9 | 0,0 | 4,4 | 1,4 | 5,1 | 4,2 | 5,2 |
| *Timon lepidus* | 10 | Med. | 0,96 | 0,99 | 8,6 | 59,8 | 0,0 | 1,0 | 0,0 | 0,1 | 0,0 | 2,1 | 0,1 | 28,3 | 0,0 |
| *Vipera seoanei* | 54 | non-Med. | 0,6 | 0,83 | 21,7 | 3,2 | 8,8 | 8,9 | 12,7 | 0,0 | 15,8 | 3,0 | 0,3 | 20,9 | 4,7 |
| *Zootoca vivipara* | 48 | non-Med. | 0,87 | 0,85 | 4,8 | 15,1 | 48,7 | 11,9 | 0,6 | 10,1 | 1,5 | 0,0 | 0,7 | 6,4 | 0,3 |
